# Transcriptome of pleuropodia from locust embryos supports that these organs produce enzymes enabling the larva to hatch

**DOI:** 10.1186/s12983-019-0349-2

**Published:** 2020-01-16

**Authors:** Barbora Konopová, Elisa Buchberger, Alastair Crisp

**Affiliations:** 1 Department of Zoology, University of Cambridge, Cambridge, UK; 2 Department of Evolutionary Developmental Genetics, University of Göttingen, Göttingen, Germany; 3 Institute of Entomology, Biology Centre of the Czech Academy of Sciences, České Budějovice, Czech Republic; 4 Department of Developmental Biology, University of Göttingen, Göttingen, Germany; 5 MRC Laboratory of Molecular Biology, Cambridge, UK

**Keywords:** Appendage, Cuticle, Ecdysone, Embryo, Gland, Immunity, Moulting fluid, Orthoptera, RNA-seq, *Schistocerca gregaria*

## Abstract

**Background:**

Pleuropodia are limb-derived glandular organs that transiently appear on the first abdominal segment in embryos of insects from majority of “orders”. They are missing in the genetic model *Drosophila* and little is known about them. Experiments carried out on orthopteran insects 80 years ago indicated that the pleuropodia secrete a “hatching enzyme” that digests the serosal cuticle to enable the larva to hatch, but evidence by state-of-the-art molecular methods is missing.

**Results:**

We used high-throughput RNA-sequencing to identify the genes expressed in the pleuropodia of the locust *Schistocerca gregaria* (Orthoptera). First, using transmission electron microscopy we studied the development of the pleuropodia during 11 stages of the locust embryogenesis. We show that the glandular cells differentiate and start secreting just before the definitive dorsal closure of the embryo and the secretion granules outside the cells become more abundant prior to hatching. Next, we generated a comprehensive embryonic reference transcriptome for the locust and used it to study genome wide gene expression across ten morphologicaly defined stages of the pleuropodia. We show that when the pleuropodia have morphological markers of functional organs and produce secretion, they are primarily enriched in transcripts associated with transport functions. They express genes encoding enzymes capable of digesting cuticular protein and chitin. These include the potent cuticulo-lytic Chitinase 5, whose transcript rises just before hatching. Unexpected finding was the enrichment in transcripts for immunity-related enzymes. This indicates that the pleuropodia are equipped with epithelial immunity similarly as barrier epithelia in postembryonic stages.

**Conclusions:**

These data provide transcriptomic support for the historic hypothesis that pleuropodia produce cuticle-degrading enzymes and function in hatching. They may also have other functions, such as facilitation of embryonic immune defense. By the genes that they express the pleuropodia are specialized embryonic organs and apparently an important though neglected part of insect physiology.

## Background

**Fig. 1:**
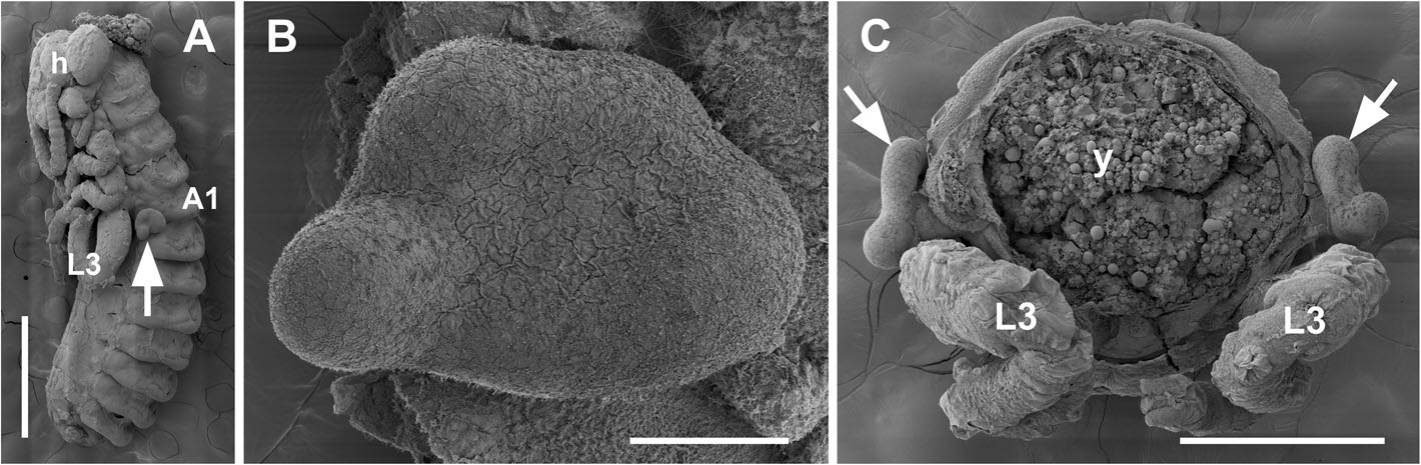
External morphology of fully developed pleuropodia in embryos of a locust *Schistocerca gregaria*. Scanning electron microscopy (SEM) images show pleuropodia that have just reached final cellular differentiation, stage 8 days in Fig. 2. (**a**) Whole embryo, yolk was removed. (**b**) Enlarged left pleuropodium. (**c**) Cross section through A1. Pleuropodium is marked with an arrow. A1, the first abdominal segment; h, head; L3, hind (third) leg; y, yolk. Scale bars: in (**a**), 1 mm; in (**b**), 100 μm; in (**c**); 500 μm

An integral part of insect embryogenesis is the transient appearance of enigmatic glandular organs on the first abdominal segment (A1) that are called pleuropodia [1, 2] (Fig. 1a-c). While many temporary structures of animal embryos have no use the pleuropodia appear to be functional organs. What exactly their function is remains uncertain. The pleuropodia originate by a peculiar modification of a pair of limb buds [3–5] and form external vesicles in some species while in others they sink down into the body wall (reviewed in e.g., [2, 6, 7]). The pleuropodia have been found in at least some species of nearly all insect “orders”, but are absent in some, like Diptera, Hymenoptera and advanced Lepidoptera such as silkworms (e.g., [5–29]). Perhaps because the pleuropodia are missing in larvae and do not develop in the genetic model *Drosophila*, they have been neglected in recent decades. The genes expressed during the active stages of pleuropodia are unknown.

Eighty years ago Eleanor Slifer [30, 31] demonstrated that the pleuropodia of the grasshopper *Melanoplus differentialis* are necessary for the digestion of serosal cuticle (SC) before hatching. SC is a chitin and protein-containing sheet structurally similar to larval and adult cuticles and is produced by extraembryonic serosa in early embryogenesis [32, 33]. SC makes a layer just under the chorion and forms a sac-like structure around the embryo and yolk. Shortly before hatching the inner layer of SC (procuticle), which forms the major part of the cuticle, disappears. When Slifer [30] removed pleuropodia from embryos, SC remained thick and the hatching larva could not break through it to get out of the egg. She proposed that pleuropodia secrete the “hatching enzyme”. The exact molecular composition of this substance is unknown, but we may assume that it is similar to the cuticle degrading moulting fluid (MF) that is released by larval epidermis under the old cuticle when the insect is preparing to moult [34].

In a few insects the ultrastructure of the cells in the pleuropodia was examined by transmission electron microscopy (TEM). These studies showed that the organs are primarily formed by an epithelium with morphological features of transporting and secretory epithelia [20, 25, 35–39]. In some insects, including orthopterans, the cells produce some secretion, but it is not clear if this is equivalent to the “ecdysial droplets” [40] carrying the MF. Some of Slifer’s experiments [30] were successfully repeated on other orthopterans [41] and an extract from pleuropodia was capable of digesting pieces of SC [42], but validation by genetic methods, such as that the pleuropodia express genes for cuticle-degrading enzymes, is missing.

Endocrinologists Novak and Zambre [43] questioned Slifer’s conclusions by arguing that according to her hypothesis SC would be digested in an unusual way compared to a typical larval cuticle. During larval moulting epidermal cells deposit a cuticle and subsequently the same epidermal cells, not a special gland, secrete MF. Therefore, they [43] proposed that the SC degrading enzymes would most probably be secreted by the serosa itself. Based on their studies in the locust (swarming grasshopper) *Schistocerca gregaria*, they suggested that the pleuropodia reach the peak of their activity in young embryos during katatrepsis and stimulate the serosa, to secrete the “hatching enzyme”. At katatrepsis the serosa is still present, but from that on it starts to shrink and completely degenerates by the time of dorsal closure in mid-embryogenesis [44]. Novak and Zambre [43] proposed that the stimulating substance released by the pleuropodia is likely a small molecule with the properties of the moulting hormone ecdysone or ecdysone itself and they carried out experiments in support of that. When a homogenate from pleuropodia was injected into the abdomen of the final instar larva of *Drosophila* that had been isolated (by a ligature) from the ecdysone producing prothoracic gland, the cuticle darkened like at pupariation. A similar effect was achieved by injection of ecdysone.

As a first step towards understanding the function of the pleuropodia we identified the genes that they express. We chose *S. gregaria* as a model, because first, it has large embryos (eggs > 7 mm long) and external pleuropodia that can easily be dissected out, second, previous experimental studies addressing the function of pleuropodia were carried out in orthopterans, and third, it is a model pest that is also used for physiological and developmental-genetic studies. Using TEM we were able to examine 11 developmental stages of the pleuropodia at high resolution. This helped us to identify when exactly the organs are fully developed and produce secretion. No detailed staging system using TEM exists for the pleuropodia of *S. gregaria* or any other orthopteran. Using high-throughput RNA sequencing (RNA-seq) we sequenced transcriptomes of ten morphologically defined stages of the pleuropodia and similarly aged hind legs and performed differential gene expression analysis between the two appendages. This enabled us to identify the transcripts enriched in the pleuropodia. The leg was chosen for comparison because the pleuropodium is a modified leg. The goal of this paper was to investigate whether the gene expression profile of the fully developed and secreting pleuropodia supports that these organs produce the “hatching enzyme”. We show that the pleuropodia of *S. gregaria* indeed express genes for some of the cuticle degrading enzymes previously identified in the MF. This brings a trancriptomic support for the Slifer’s historic hypothesis [30, 31]. As a part of our study we assembled a full embryonic transcriptome of *S. gregaria*, whose genome has not been sequenced yet.

## Results

### Development of pleuropodia in the course of *S. gregaria* embryogenesis

**Fig. 2:**
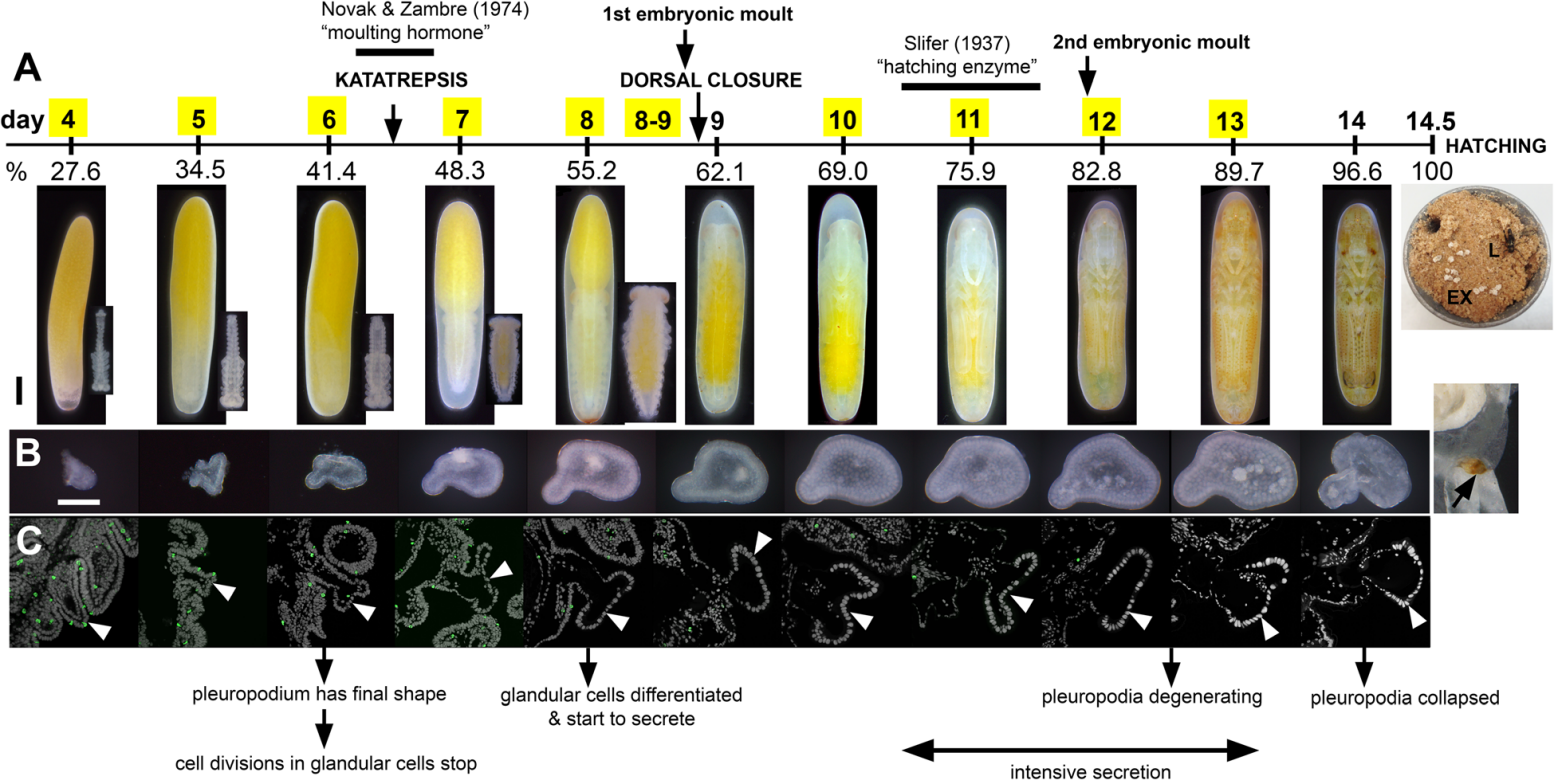
Summary of the development of pleuropodia in *S. gregaria* embryos. (**a**) Scheme of *S. gregaria* embryogenesis marking key developmental events in the embryos and timing of the two experiments on pleuropodia. Numbers above the scale are days from egg-laying, numbers below the scale are percent of embryonic developmental time. Yellow boxes indicate the stages that were sampled for RNA-seq. Eggs with the developing embryos at each stage are shown below the scale, insets for the 4–8 day stages show the embryo dissected out from the egg. (**b**) External features of the developing pleuropodia; after hatching part of the stretched exuvia is shown; the degenerated pleuropodium is marked with an arrow. (**c**) Paraffin sections through the pleuropodium and surrounding tissue. Pleuropodia are marked with arrowheads. Anti-Phospho-Histone H3 antibody (green) detects cell divisions in the immature glandular cells (tip of appendage bud) on day 4 and 5, not in later stages. The pleuropodial stalk cells, haemocytes entering the pleuropodia and cells in other tissues were labeled. Nuclei (grey) enlarge from day 6. The text below the pictures refers to the main events in the glandular cells. EX, exuvia; L, larva. Scale bars: in (**a**) (eggs), 1 mm; in (**b**), 0.2 mm. Background was cleaned in photos in (**a**) (see Methods)

Under our incubation conditions (see Methods) *S. gregaria* embryogenesis lasts 14.5 days (100 % developmental time, DT) (Fig. 2a, Additional file 1: Figure S1). We followed the development of the pleuropodia from the age of 4 days (27.6 % DT), when all appendages are similar looking short buds, until just before hatching, day 14 (Fig. 2b, Additional file 1: Figures S2-S3). Simultaneously, we followed the development of the hind leg, which we used for comparison.

We traced cell divisions in the pleuropodia by using Phospho-Histone H3 as a marker (Fig. 2c). The glandular cells were labeled only in the days 4 and 5. From day 6 onwards no cell divisions were detected and the nuclei started to enlarge as the cells became polyploid [45].

**Fig. 3:**
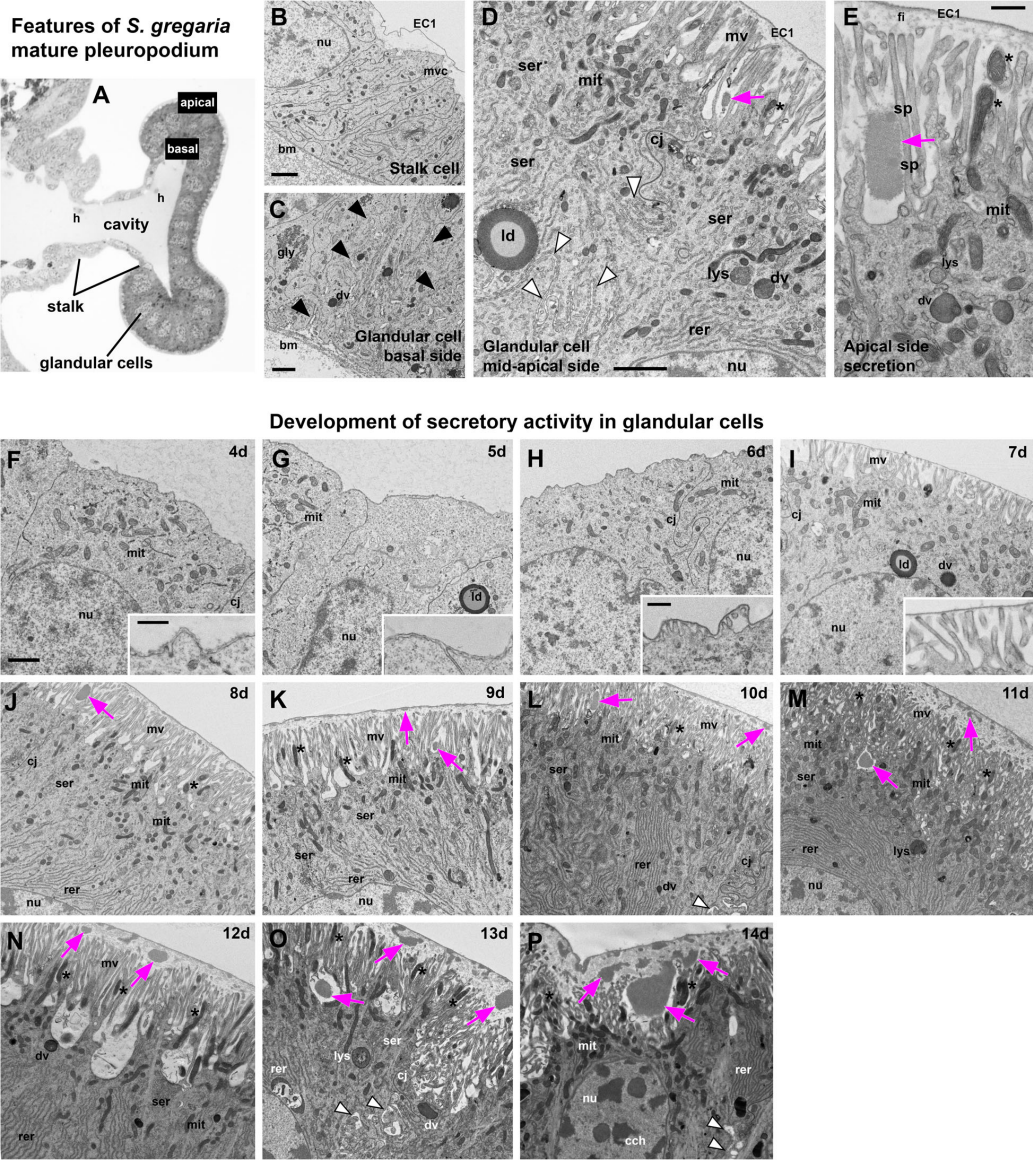
Ultrastructure and development of secretory activity in pleuropodia of *S. gregaria*. (**a**)-(**e**) Main features of the cells in the fully formed pleuropodia. Pleuropodia just before dorsal closure are shown: (**a**-**d**), 8 days; (**e**), 8.5 days. (**a**) Cross section through the pleuropodium. Apical (“outer”) and basal (“inner”) side of the cells is labeled. (**b**) Stalk cell. The short microvilli at the apical side are associated with the deposition of fibres in the embryonic cuticle (EC1). (**c**)-(**e**) Glandular cells. Note in (**e**) that the secretion granule is located at the base of the microvilli (brush-border); the tips of the microvilli produce fibrous material that is a part of the embryonic cuticle (EC1). (**f**)-(**p**) Ontogenesis of the glandular cells. Note the development of the microvilli, which is for day 4–7 shown at higher magnification in insets (**f**)-(**i**) (compare with fully developed microvilli shown at high magnification in (**d**) and (**e**)), accumulation of mitochondria below, mitochondria entering the microvilli, presence of smooth and rough endoplasmic reticula and the onset of secretion (appearance of secretion granules within and above the microvilli). (**a**) is a toluidine blue stained semithin section, (**b**)-(**p**) TEM micrographs. Secretion granules are marked with magenta arrows. Asterisks mark mitochondria inside microvilli. Black arrowheads mark the infolding of the basal plasma membrane (basal labyrinth) shown in (**c**), white arrowheads mark the spaces between neighboring cells. bm, basement membrane; cch, condensed chromatin; cj, cell junction; dv, dense vesicle; EC1, the first embryonic cuticle; gly, glycogene; ld, lipid droplet; mit, mitochondria; mv, microvilli; nu, nucleus; rer, rough endoplasmic reticulum; ser, smooth endoplasmic reticulum; sp., “spot” of a different electron-density in the pleuropodial granules. Scale bars: in (**b**), (**c**), (**d**), (**e**) and (**f**) for (**f**)-(**p**), 2 μm; inset in (**f**) for inset in (**f**) and (**g**), 500 nm, inset in (**h**) for inset in (**h**) and (**i**), 500 nm

Although the pleuropodia get their final external mushroom-like shape just before the embryos undergo katatrepsis (day 6; 41.4 % DT) (Fig. 2a,b), (Fig. 3) the glandular cells fully differentiate only later, shortly before dorsal closure (day 8; 55.2 % DT). The differentiated cells (Fig. 3c-e, j-p), compared to the immature ones (Fig. 3f-i) have fully developed apical microvilli, abundant mitochondria below the microvilli and some inside the microvilli, developed rough and smooth endoplasmic reticula. The cells form a single-layered epithelium and secretion granules inside and outside of them become visible (Fig. 3a-e, j). The granules outside of the cells first appear at the base and in between the long apical microvilli (brush-border) (Fig. 3e,j). Similarly as it has been observed in other insects [25, 35–39], the whole pleuropodium is covered with a thin embryonic cuticle (“the first embryonic cuticle”, EC1); the tips of the microvilli produce fibrous material that is a part of this cuticle (Fig. 3e) (compare with similar fibers above the leg epidermis in Additional file 1: Figure S4).

As development progresses the secretion granules (inside and outside the cells) become more abundant and are present also above the microvilli (Fig. 3k-p). On day 12 the apical side of the glandular cells changes: clusters of microvilli (usually at the borders between cells) elevate (Fig. 3n). Later the cells show signs of degeneration, the chromatin condenses and the cell content becomes disorganized (Fig. 3o,p). Large secretion granules are still abundant and probably released even on the last day before hatching, when the pleuropodia have shrunk and collapsed (Figs. 2b,3p).

**Fig. 4:**
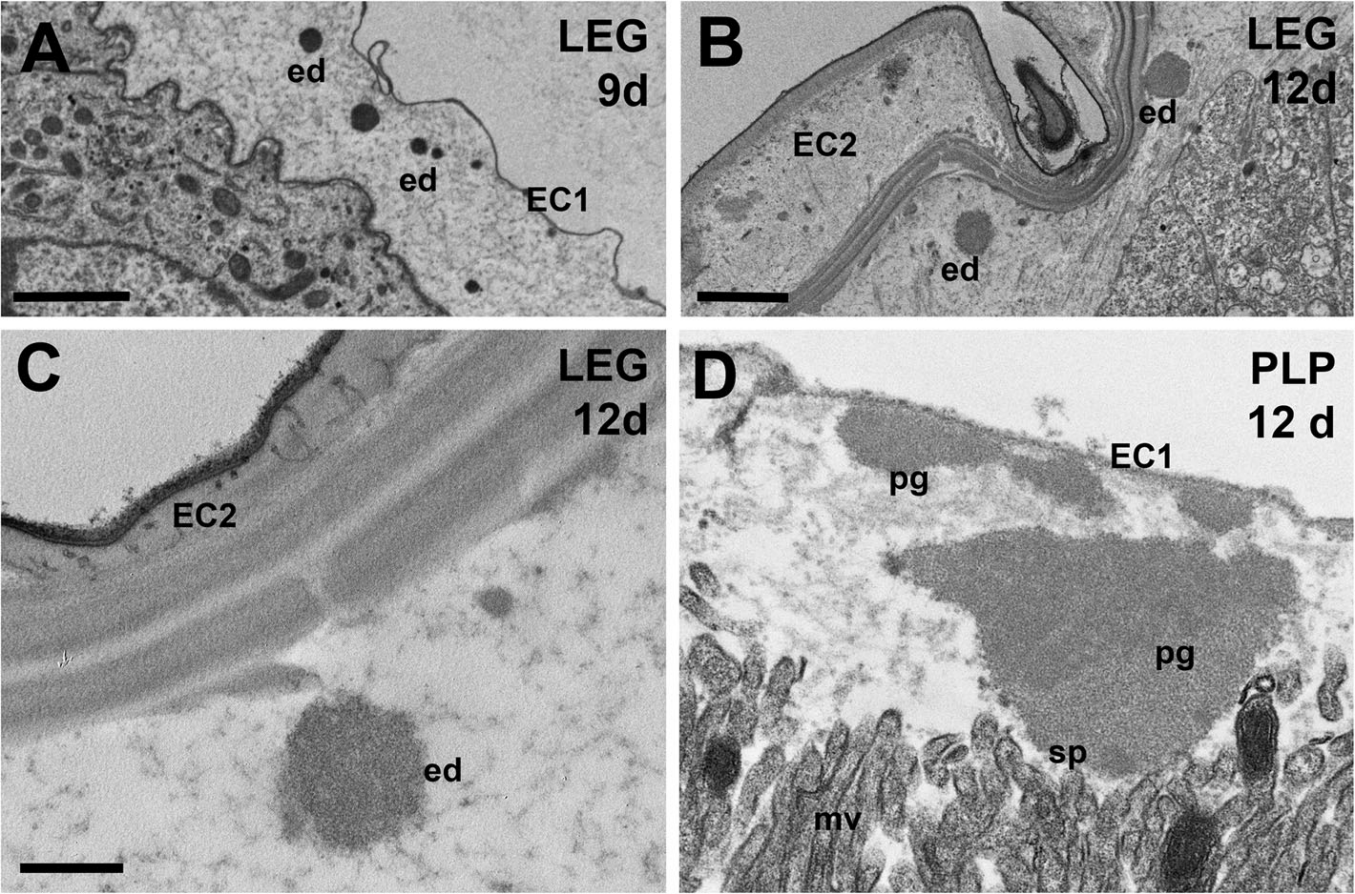
Granules secreted from pleuropodia of *S. gregaria* resemble ecdysial droplets. Release of ecdysial droplets by embryonic epidermis on a hind leg during the first (**a**) and the second (**b**) embryonic moult, on day 9 and day 12, respectively. (**c**) Ecdysial droplet secreted during the second embryonic moult at higher magnification and (**d**) granules secreted from pleuropodia at the same developmental stage. The pleuropodial granules are typically larger, less compact and with non-homogeneous electron density. EC1, EC2, the first and second embryonic cuticles; ed, ecdysial droplets; mv, microvilli; pg, granules secreted from the pleuropodia; PLP, pleuropodium; sp, “spot” of a different electron-density in the pleuropodial granules. Scale bars: in (**a**) and (**b**), 2 μm; in (**c**) for (**c**) and (**d**), 500 nm

When the embryo moults (embryonic cuticle that had been deposited by the epidermal cells of the embryo detaches from these cells, which then secrete a new cuticle), first at about 8.5 days and again just before 12 days (Fig. 2a, Additional file 1: Figure S4), ecdysial droplets are present below the apolysed cuticle. These droplets are very similar at both moults (compare Fig. 4a and b; also shown in Additional file 2: Figure S4f and i). They are very similar, but not identical to the granules released by the pleuropodia (compare Fig. 4c and d). The glandular cells of the pleuropodia do not moult and keep the cuticle EC1 their whole life-time.

At hatching, the larva enclosed in the apolysed second embryonic cuticle (EC2) leaves the eggshell and digs through the substrate up to the surface [46, 47]. Here the EC2 is shed and the degenerated pleuropodia are removed with it ([7]; Fig. 2a).

Therefore, the timing of the high secretory activity (from ±69 % DT until just before hatching) corresponds to the stages when Slifer [30] demonstrated the presence of the “hatching enzyme” (Fig. 2a). By contrast, pleuropodia from embryos around katatrepsis (± 41–48 % DT) that Novak and Zambre [43] used for their experiments do not appear fully differentiated and secretory yet. Next we looked at the genes expressed in the pleuropodia.

### Isolation of genes expressed in the pleuropodia of *S. gregaria* using RNA-seq

Prior to the gene expression analysis we prepared a comprehensive embryonic transcriptome that served as a reference (see Methods). It consists of 20,834 transcripts (Additional file 2: Table S1) and we aimed each transcript to represent one gene. The completeness of the transcriptome was assessed using the open-source software BUSCO (version 3) [48, 49]. 95.6, 96.3 and 94.6 % of the Metazoa, Arthropoda and Insecta orthologs, respectively, were found, a level comparable to published “complete” transcriptomes.

**Fig. 5:**
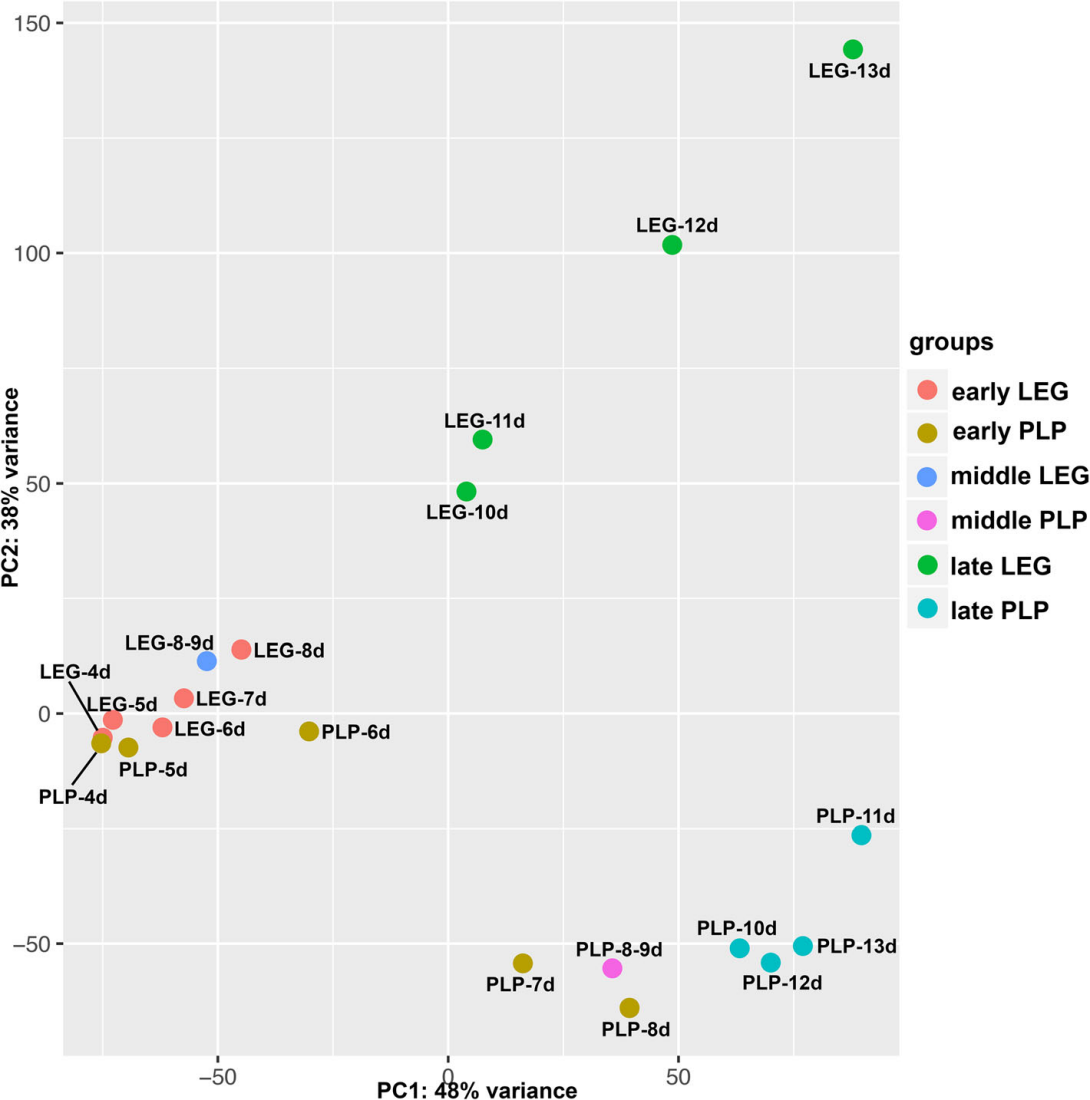
Principal component analysis (PCA) plot on genes expressed in legs and pleuropodia at ten embryonic stages. Samples from young embryos are genetically more similar and cluster together, while samples from advanced stages are genetically more distant and also separated on the plot. Rlog transformed read counts

We generated a comparative RNA-seq dataset from ten stages of developing pleuropodia and hind legs (Fig. 2a). We dissected pleuropodia and legs from the embryos and sequenced their mRNAs. For each stage we performed a differential gene expression analysis between a sample from pleuropodia and a sample from legs (Fig. 2a, Additional file 2: Table S2). A principal component analysis (PCA) confirmed that pleuropodia and legs are not only morphologically similar at early stages, but share a transcriptomic landscape as well (Fig. 5a). The expression profiles diversify as the appendages progressively develop into completely different structures. The number of differentially expressed genes (DEGs) rises with age (Additional file 2: Table S3). For genes whose expression dynamics in the pleuropodia are known, such as *Ubx*, *abd-A*, *dll* and *dac* (e.g., [3, 50–54]), we confirmed that they were up- or downregulated in our RNA-seq dataset as predicted (Additional file 2: Table S4). To further validate the dataset, we carried out real-time RT-PCR on 46 selected genes in several stages (176 cases in total) and got results consistent with the sequencing data (Additional file 2: Table S5).

Since we here focus on the secreting pleuropodia we pooled the data from samples 10, 11 and 12 days together (pleuropodia and legs separately), and treated them as triplicates. These are samples from embryos after dorsal closure, when secretion granules are highly abundant above the pleuropodial cells, but the organs are not in advanced state of degeneration yet (day 13) (Figs. 2a, 3l-n). We found 781 transcripts upregulated and 1535 downregulated in pleuropodia (compared to legs) (Additional file 2: Table S3). Table 1 shows the top 10 % of the most highly abundant (“expressed”) upregulated transcripts (abundance measured in RPKM units, “reads per kilobase of transcript per million reads mapped”).

**Table 1:** Top 10 % of the most abundant transcripts upregulated in the highly secreting pleuropodia

Transcript ID	Protein	Characteristics	Immunity^a^	Cuticle digestion^b^	RPKM		Fold change
					legs	pleuropodia	
SgreTa0017702	-				23.07	15,186.05	658.36
SgreTa0007897	C-type lysozyme	anti-bacterial protein	x		42.93	14,452.15	336.64
SgreTa0002988	Uncharacterized, contains DUF4773 domain				15.16	9112.05	601.19
SgreTa0005052	-				13.37	7950.98	594.48
SgreTa0001636	Serine protease	proteolysis	x	x	49.38	7578.31	153.48
SgreTa0008851	Chitin binding Peritrophin-A	perotrophic matrix protein			9.12	6836.42	749.88
SgreTa0017707	I-type lysozyme	anti-bacterial protein	x		12.20	6712.31	550.26
SgreTa0007042	-				7.04	6650.18	944.25
SgreTa0004599	Alpha-tocopherol transfer protein	intermembrane lipid transfer			8.99	5848.12	650.71
SgreTa0009217	-				5.03	5384.56	1070.14
SgreTa0003175	Collagen				32.25	5220.96	161.87
SgreTa0007886	Alpha-N-acetylgalactosaminidase	carbohydrate catabolism			3.85	4372.63	1134.69
SgreTa0002109	-				2.20	3016.31	1372.07
SgreTa0017715	Serine protease, Snake-like	proteolysis, Toll signaling	x	x	70.55	2947.46	41.78
SgreTa0017664	Chitinase 5	cuticular chitin degradation		x	79.32	2620.11	33.03
SgreTa0002467	Neutral endopeptidase 24.11	proteolysis		x	62.26	2282.01	36.66
SgreTa0004397	-				11.21	2266.30	202.21
SgreTa0002828	-				1.77	2188.14	1234.00
SgreTa0006539	Serpin, 88E-like	serine protease inhibitor	x		32.42	2152.14	66.38
SgreTa0001321	Glycosyl hydrolase, Myrosinase 1-like	carbohydrate catabolism			3.93	2070.40	527.16
SgreTb0011177	-				1.38	1884.79	1369.32
SgreTa0008335	-				54.24	1812.38	33.41
SgreTa0003635	Alpha-tocopherol transfer protein	intermembrane lipid transfer			2.23	1800.68	806.99
SgreTb0003860	Serine protease, H2-like	proteolysis	x	x	77.42	1727.41	22.31
SgreTa0013418	-				0.87	1484.98	1710.66
SgreTa0014009	Angiotensin-converting enzyme	proteolysis		x	65.76	1457.47	22.16
SgreTa0006966	Pro-phenol oxidase subunit 2	immunity, melanization	x		144.78	1347.43	9.31
SgreTa0000425	6-phosphofructo-2-kinase	glycolysis			93.52	1346.50	14.40
SgreTa0003661	Serine protease, Easter-like	proteolysis	x	x	29.50	1332.79	45.18
SgreTa0006960	Glutamate dehydrogenase mitochondrial	nitrogen and glutamate metabolism			172.56	1327.45	7.69
SgreTa0017670	Xaa-Pro aminopeptidase	proteolysis		x	2.89	1322.01	457.96
SgreTb0000759	Cathepsin L	proteolysis, lysosomal enzyme		x	105.63	1308.36	12.39
SgreTa0014684	-				1.30	1294.87	994.80
SgreTa0007025	Insect pheromone-binding protein A10/OS-D	chemoreception			1.77	1224.20	692.95
SgreTa0006282	Cytochrome P450 CYP4G102	synthesis of hydrocarbons, anti-dehydration			2.91	1196.27	410.93
SgreTa0009515	Sensory neuron membrane protein, 1-like	chemoreception			3.33	1188.81	357.50
SgreTa0008528	C-type lysozyme	anti-bacterial protein	x		8.61	1159.55	134.71
SgreTa0009095	Catalase	redox homeostasis	x		355.15	1158.27	3.26
SgreTb0039135	-				3.53	1119.22	316.71
SgreTa0001486	Lipopolysaccharide-induced tumor necrosis factor-alpha factor homolog	lysosomal degradation			45.83	1109.33	24.20
SgreTb0039012	-				14.29	1060.82	74.25
SgreTa0009747	Serpin (27-like)	serine protease inhibitor, melanization	x		14.49	1054.67	72.80
SgreTa0013400	Peroxiredoxin, 5-llke	redox homeostasis	x		101.10	1034.15	10.23
SgreTa0017395	-				5.08	1004.86	197.64
SgreTa0017712	-				15.59	990.41	63.53
SgreTa0005600	Beta-N-acetylglucosaminidase NAG2	cuticular chitin degradation		x	15.10	939.60	62.21
SgreTa0000783	Serine protease, Snake-like	proteolysis	x	x	4.30	917.47	213.59
SgreTa0006651	Uncharacterized, contains Transcription activator MBF2 domain				1.62	907.98	561.49
SgreTa0017657	Putative serine protease, K12H4.7-like / Serine carboxypeptidase	proteolysis		x	2.31	904.26	391.60
SgreTa0017700	Peroxidase	redox homeostasis	x		5.36	874.51	163.25
SgreTa0002600	Uncharacterized, contains DUF3421 domain				0.97	870.73	894.35
SgreTb0019827	Tob	antiproliferative protein, transcription corepressor			141.26	846.86	5.99
SgreTa0017854	-				0.85	838.89	981.74
SgreTa0007774	Lysosomal-associated membrane protein	lysosomal membrane protein			185.20	822.81	4.44
SgreTa0015156	-				27.45	804.82	29.32
SgreTa0007809	Tetraspanin	scaffolding protein in cell membrane			63.04	799.76	12.69
SgreTa0004471	Leucine rich repeat	membrane glycoprotein			74.88	797.35	10.65
SgreTa0004278	Fatty acyl-CoA reductase, waterproof-like	lipid metabolism			1.75	733.39	417.99
SgreTa0014626	V-type proton ATPase proteolipid subunit	proton transporting ATPase			190.76	708.56	3.71
SgreTa0016256	Bax inhibitor 1	negative regulation of apoptosis and autophagy			237.58	692.52	2.91
SgreTa0001469	Sodium/potassium-transporting ATPase subunit alpha	sodium:potassium exchanging ATPase			119.60	685.51	5.73
SgreTa0007426	Serine protease, Easter-like	proteolysis	x	x	0.66	673.43	1023.60
SgreTa0007081	Vigilin	RNA binding, sterol metabolism			247.46	655.61	2.65
SgreTa0013328	Ferritin	iron ion transport, iron sequestration	x		238.10	651.31	2.74
SgreTa0002155	Uncharacterized serine protease inhibitor	serine protease inhibitor	x		33.83	646.73	19.12
SgreTa0014303	-				176.21	645.78	3.66
SgreTa0017577	Aquaporin	water channel			0.39	635.34	1638.96
SgreTa0013377	Phosphoenolpyruvate carboxykinase [GTP]	gluconeogenesis			13.56	628.95	46.37
SgreTa0005752	Alpha-tocopherol transfer protein	intermembrane lipid transfer			12.98	594.56	45.79
SgreTa0014098	Phospholipase B-like	lipid degradation			206.76	577.99	2.80
SgreTa0000856	Transposase-like				25.93	576.67	22.24
SgreTa0008861	-				0.37	541.63	1456.67
SgreTa0017826	Sodium:neurotransmitter symporter	solute:sodium symport			0.49	540.53	1104.10
SgreTb0019287	-				3.11	528.47	169.79
SgreTa0015520	Protein yellow	melanization	x		2.75	520.09	189.08
SgreTb0006243	I-type lysozyme	anti-bacterial protein	x		16.96	519.35	30.62
SgreTa0009559	Gram-negative bacteria binding protein 3	pathogen recognition	x		15.40	510.04	33.13

^a^ proteins related to immune response

^b^ proteins that participate in larval moulting; some of them are known, other anticipated to digest cuticular chitin and protein (e.g., present in the MF)

**Fig. 6:**
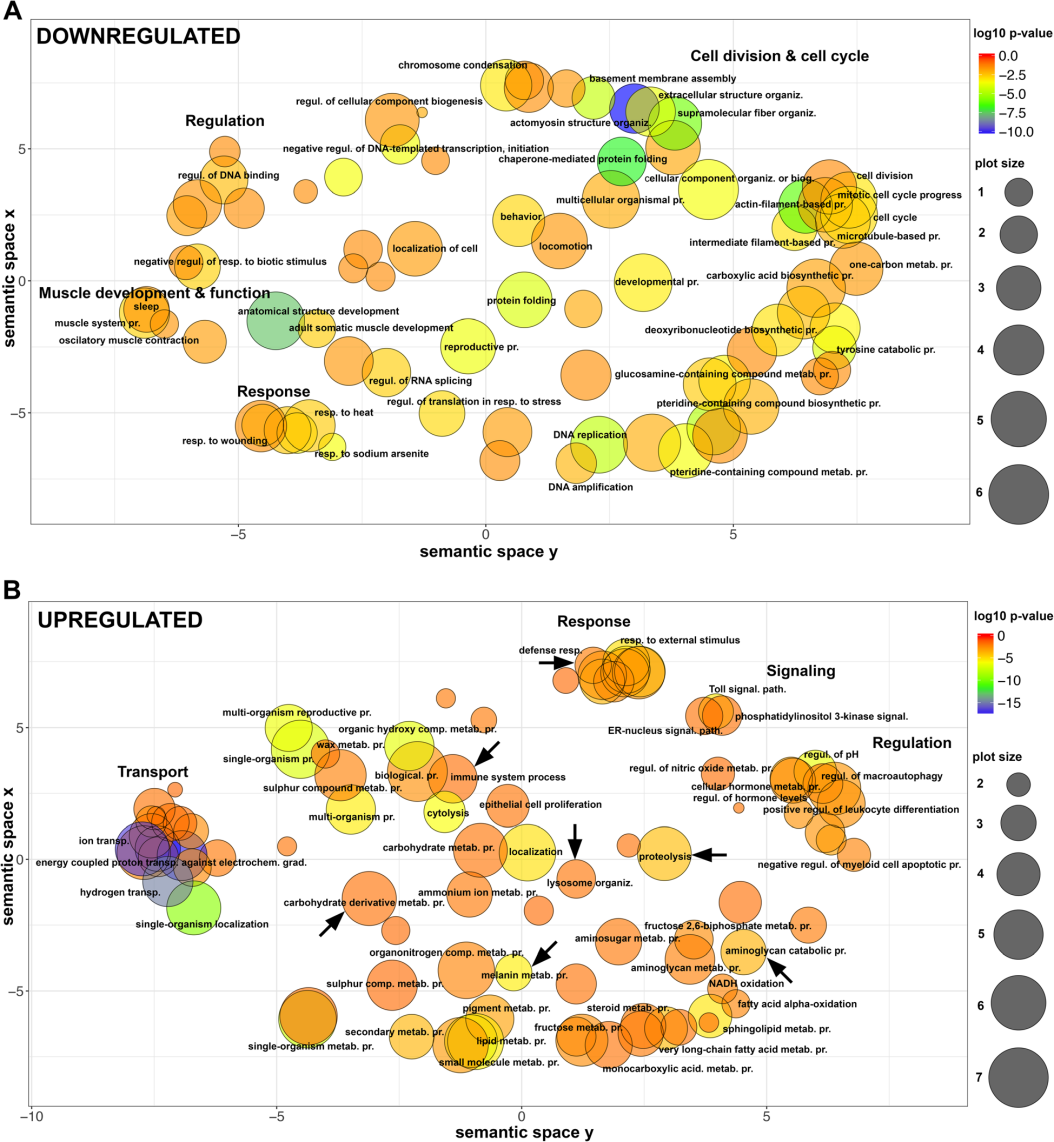
Dot plot visualization of GO terms enriched in DEGs in the highly secreting pleuropodia. Representative groups of GO terms enriched in genes that are (**a**) downregulated in pleuropodia (in comparison to legs) and (**b**) upregulated in pleuropodia. Major clusters are labeled. Relevant GOs are marked with an arrow. Bubble color indicates the *p*-value of the GO term, the size indicates the frequency of the GO term in the underlying Gene Ontology Annotation (GOA) database (bubbles of more general terms are larger). biog., biogenesis; comp., compound; electrochem., electrochemical; grad., gradient; metab., metabolic, organiz., organization; path., pathway; pr., process; regul., regulation; resp., response; signal., signaling; transp., transport

GO enrichment analysis, graphically summarized in Fig. 6 (full set of enriched GO terms are in Additional file 2: Tables S6, S7; GO terms enriched at each developmental stage are in Additional file 2: Tables S8, S9), showed that the genes downregulated in pleuropodia, thus upregulated in legs, are enriched in GO terms associated with development and function of muscles and cell division. This is consistent with the absence of muscles in the pleuropodia that are fully differentiated organs at this stage, while the legs are growing in size and developing muscle tissue (Fig. 2c).

The upregulated genes are primarily enriched in GO terms (Fig. 6, Additional file 2: Table S7) associated with active transport, which is consistent with that the pleuropodia have morphological characteristics of transporting organs. Genes for both V-ATPase and Na^+^, K^+^ ATPase are upregulated (Additional file 2: Table S10). We found enriched GO terms linked with lysosome organization, consistent with the observation that the pleuropodia contain numerous lysosomes (Fig. 3, [37]). We also found a cluster of GO terms associated with lipid metabolism, consistent with the presence of smooth endoplasmic reticulum in the cells (Fig. 3). Therefore, the genes differentially expressed between legs and pleuropodia are in agreement with the morphology of the organs.

Insect cuticle, such as SC, is digested by a cocktail of enzymes that degrade chitin, which is an aminoglycan polymer, and proteins [34, 55, 56]. In support of Slifer’s experiments demonstrating that pleuropodia produce cuticle degrading enzymes we found that these organs upregulate genes associated with carbohydrate derivative metabolism, aminoglycan catabolic process and proteolysis. A novel interesting finding was the upregulation of genes associated with immunity. Next we looked at particular genes in a detail.

### Pleuropodia upregulate genes for cuticular chitin degrading enzymes

Cuticular chitin is hydrolyzed by a two-enzyme system composed of a β-N-acetyl-hexosaminidase (NAG) and a chitinase (CHT) [56]. Both types of enzymes, a NAG and a CHT, have to be simultaneously present for efficient hydrolysis of chitin [57].

**Fig. 7:**
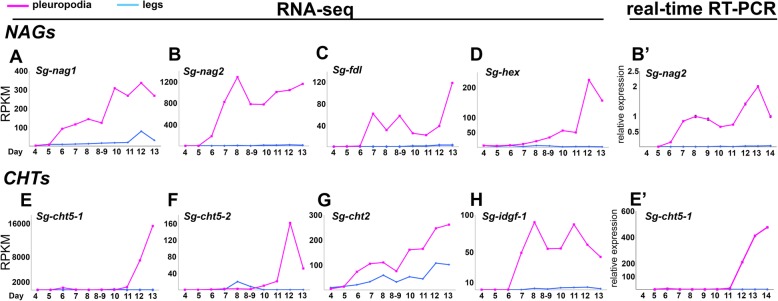
Expression profiles of NAGs and CHTs upregulated in the pleuropodia of *S. gregaria* across development. Top row: NAGs, bottom row: CHTs. (**a**)-(**d**) and (**e**)-(**h**): RNA-seq; expression in single-sample sequencing is shown. (**b**') and (**e**'): real-time RT-PCR. (**b**') is the same gene as in (**b**) and (**e**') is the same gene as in (**e**). Analysis of 3–4 technical replicates is shown. Expression in day 8 was set as 1. Values are mean ± s.d.

Insect NAGs split into four major classes, of which chitinolytic activity was demonstrated for group I and II (Table 2) [58, 59]. Our transcriptome contains four NAG transcripts, each representing one group (Table 2, Fig. 7a-d, Additional file 1: Figure S5a, S6a). All were upregulated in the pleuropodia. Among them the *Sg-nag2* for the chitinolitic NAG group II had the highest expression (among 46 most highly “expressed” genes, Table 1) and fold change between legs and pleuropodia. The abundance of transcripts for the chitinolitic NAGs starts to rise from day 6 (Fig. 7a,b) when the glandular cells in the pleuropodia begin to differentiate morphologically (Figs. 1 and 3). The expression profile of *Sg-nag2*, that we have chosen for validation, was similar by RNA-seq and real-time RT-PCR (compare Fig. 7b and b').

**Table 2:** RNA-seq differential gene expression of cuticular chitin-degrading enzymes in the highly secreting pleuropodia

Family	Group	Protein	*S. gregaria* gene	UP/DOWN^a^	Fold change	Expression^b^
β-N-acetylhexosaminidase	I	NAG1	***Sg-nag1***	**UP**	**7.85**	**124 (15.88 %)**
	II	NAG2	***Sg-nag2***	**UP**	**62.21**	**46 (5.89 %)**
	III	Fused lobes	*Sg-fdl*	UP	14.18	592 (75.8 %)
	IV	Hex	*Sg-hex*	UP	47.37	306 (39.18 %)
Chitinase	I-Major "moulting" chitinases	Chitinase 5	***Sg-cht5-1***	**UP**	**33.03**	**15 (1.92 %)**
			*Sg-cht5-2*	UP	234.78	400 (51.21 %)
	II-"Moulting" chitinases	Chitinase 10	*Sg-cht10-1*	na^c^		
			*Sg-cht10-2*	ns^d^		
	III-Cuticle assembly chitinases	Chitinase 7	*Sg-cht7-1*	ns		
			*Sg-cht7-2*	ns		
			*Sg-cht7-3*	ns		
	IV-Gut, fat body and other chitinases	Chitinase 8	*Sg-cht8-1*	na		
			*Sg-cht8-2*	na		
			*Sg-cht8-3*	na		
		Chitinase 6	*Sg-cht6-1*	ns		
			*Sg-cht6-2*	ns		
		Chitinase 2	***Sg-cht2***	**UP**	**2.81**	**188 (24.07 %)**
	V-Imaginal disc growth factors	Idgf	*Sg-idgf-1*	**UP**	20.97	391 (50.06 %)
			*Sg-idgf-2*	ns		
			*Sg-idgf-3*	ns		

^a^ upregulated (UP)/ downregulated (DOWN)

^b^ the DEGs (781 upregulated) were ranked according to their RPKM in descending order, the number describes the position of the DEG in the ranked table; transcripts in bold were among the top 25 % most abundant

^c^ not applicable (expression low to undetectable in both samples, transcript filtered out)

^d^ not significant

**Fig. 8.**
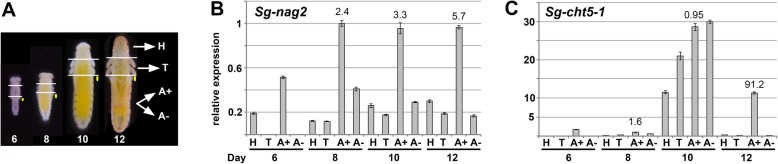
Real-time RT-PCR expression analysis of *Sg-nag2* and *Sg-cht5–1* on cDNA from parts of *S. gregaria* embryos. (**a**) cDNA was prepared from mRNAs isolated from parts of embryos at the age of 6, 8, 10 and 12 days: H, head; T, thorax; A+, abdomen with pleuropodia; A-, abdomen without pleuropodia. For each age the same number of body parts was used (5–10) and RNA was resuspended in the same volume of water. The size of the pleuropodium is indicated by the yellow dot. (**b**) and (**c**): expression of *Sg-nag2* and *Sg-cht5–1*, respectively. Analysis of 3–4 technical replicates is shown. Expression in A + 8 (abdomen with pleuropodia at stage when the organs first become differentiated) was set as 1. Numbers above A+ expression is fold change from A- of the same age

To see if the pleuropodia are the major source of the *Sg-nag2* transcript in the embryo, we looked at its expression in various parts of the body (head, thorax, abdomen with pleuropodia, abdomen from which pleuropodia were removed) using real-time RT-PCR (Fig. 8a,b). We performed this analysis in embryos on day 6, when the pleuropodia are still immature, day 8, just at the onset of the secretory activity, day 10 and day 12 during active secretion. During all of the stages the abdomen with pleuropodia had the highest expression (A+ in Fig. 8b), although the expression was lower in the youngest sample (day 6) compared to the samples from older embryos (day 8, 10 and 12). This shows that the pleuropodia are the major source of mRNAs for this cuticle-degrading NAG.

The insect CHTs have been classified into several groups [56, 60], of which the major role in the digestion of cuticular chitin is played by Chitinase 5 and (perhaps with a secondary importance) by Chitinase 10 [61, 62] (Table 2; the classification of CHTs into five major groups that we use here is based on [62]). Some chitinases, for example, are expressed in the gut, trachea and fat body, where they are likely involved in digestion of dietary chitin, turnover of peritrophic matrix and immunity, some epidermal chitinases organize assembly of the new cuticle (e.g., [60, 63–66]).

Our transcriptome contains 16 full or partial transcripts of CHTs representing all of the major CHT groups (Table 2, Additional file 1: Figure S5b, S6b). The pleuropodia specifically upregulate both of the genes for Chitinase 5, which show homology with *cht5–1* and *cht5–2* from the locust *Locusta migratoria* [67]. One of the transcripts, *Sg-cht5–1*, was among the top 15 most abundant transcripts upregulated in the highly secreting pleuropodia (Table 1). The predicted amino acid sequence of *Sg-cht5–1* contains a conserved catalytic domain and a signal peptide, and thus is likely to be active and secreted, respectively (Additional file 1: Figure S5b). The other upregulated CHTs were homologs of *cht2* and *idgf*. By contrast, the *S. gregaria* homolog of *cht10* that also has a role in cuticular chitin hydrolysis and required for larval moulting [62, 64] had low expression in both legs and pleuropodia.

We next focused on the transcript of the major chitinase, *Sg-cht5–1*. Unlike the NAGs, both RNA-seq and real-time RT-PCR have shown that the expression of this CHT is low in the early secreting stages, rises only later around day 12 and reaches highest levels when the pleuropodia are already degenerating (day 13 and 14) (Fig. 7 e,f,e'). Also real-time RT-PCR on cut embryos has shown that the pleuropodia are a major source of the *Sg-cht5–1* mRNA on day 12 but not before (the high expression in the whole embryo on day 10 could be linked to the second embryonic moult and was also observed with *Sg-cht7*, although not with *Sg-cht10*, Additional file 1: Figure S7). These data show that the pleuropodia before hatching express a cuticle-degrading chitinase.

### Pleuropodia upregulate transcripts for some proteases that could digest a cuticle

Our GO enrichment analysis has shown that the secreting pleuropodia are enriched in transcripts for genes associated with proteolysis (Fig. 6, Additional file 2: Table S11). Transcripts for proteases and their inhibitors are abundant among the top 10 % of the most highly “expressed” upregulated DEGs (Table 1). To see if the upregulated transcripts encode enzymes that are associated with digestion of insect cuticle, we compared our data with the enzymes identified in the complete proteomic analysis of the MF from the lepidopteran *Bombyx mori* [55, 68]. Out of 69 genes that we searched, we found homologs or very similar genes in *S. gregaria* transcriptome for half of them (35). This made in total 75 transcripts, of which 27 were upregulated (seven among the top 10 % most highly expressed) and 15 downregulated (Tables 3, Additional file 2: Table S12). The prominent MF protease Carboxypeptidase A [55, 69] and the Trypsin-like serine protease known to function in locust moulting [70] were not upregulated in the pleuropodia. These data indicate that the pleuropodia upregulate transcripts for proteolytic enzymes associated with the degradation of the cuticle and would be able to contribute to the digestion of SC, although the enzymatic cocktail produced by the pleuropodia may not be identical with the MF.

**Table 3:** MF proteases that were upregulated in the highly secreting pluropodia

MF protein^a^	Blast query^b^	*S. gregaria* transcript ID^c^	homolog/similar^d^	RPKM PLP	Fold change UP
Putative peptidase	D2KMR2	SgreTa0000627	similar	131.75	3.14
Aminopeptidase N-12	I3VR83	SgreTb0018983	similar	35.86	4.35
Neutral endopeptidase 24.11	Q9BLH1	**SgreTa0002467**	similar	2282.01	36.66
	Q9BLH1	SgreTa0017692	similar	133.30	240.28
	Q9BLH1	SgreTb0039123	similar	219.35	186.96
Ecdysteroid-inducible angiotensin-converting enzyme	Q9NDS8	**SgreTa0014009**	similar	1457.47	22.16
	Q9NDS8	SgreTa0017728	similar	62.71	57.08
Carboxypeptidase E-like	H9IST0	SgreTa0000925	homolog	139.81	10.95
Angiotensin-converting enzyme-like	H9IZ41	SgreTa0003298	homolog	23.64	5.65
Aminopeptidase N-like	H9JEW9	SgreTa0017219	homolog	391.03	437.93
Digestive cysteine protease 1, cathepsin L	H9JHZ1	SgreTa0000627	homolog	131.75	3.14
Serine carboxypeptidase	H9J242	**SgreTa0017657**	homolog	904.26	391.60
Serine protease HP21 precursor	H9JJA9	SgreTa0017649	similar	179.69	24.45
Trypsin-like serine protease - fibroin heavy chain	H9JPA8	**SgreTa0001636**	homolog	7578.31	153.48
Serine protease, Easter-like	Q2VG86	SgreTa0003188	homolog	485.97	837.45
	Q2VG86	**SgreTa0003661**	homolog	1332.79	45.18
	Q2VG86	SgreTa0006780	homolog	103.37	14.76
	Q2VG86	SgreTa0007424	homolog	29.62	79.13
	Q2VG86	SgreTa0007425	homolog	123.69	72.31
	Q2VG86	SgreTb0037249	homolog	21.76	249.74
	Q2VG86	SgreTb0039879	homolog	305.63	544.04
	H9JLZ4	SgreTa0010219	similar	46.12	20.75
	H9JLZ4	SgreTb0039024	similar	11.71	22.11
Serine protease 1	H9JXY6	**SgreTb0003860**	homolog	1727.41	22.31
Serine protease, Snake-like	H9IWW2	**SgreTa0000783**	similar	917.47	213.59

^a^ proteomic sequencing of MF of the lepidopteran *Bombyx mori* ([55, 68])

^b^ Uniprot ID for blast on *S. gregaria* transcriptome

^c^ transcripts in bold were among the top 10 % most highly “expressed” upregulated DEGs (Table 1)

^d^ considered as homologous, if reciprocal blast retrieved the query sequence

### Pleuropodia are enriched in transcripts for immunity-related proteins

**Fig. 9:**
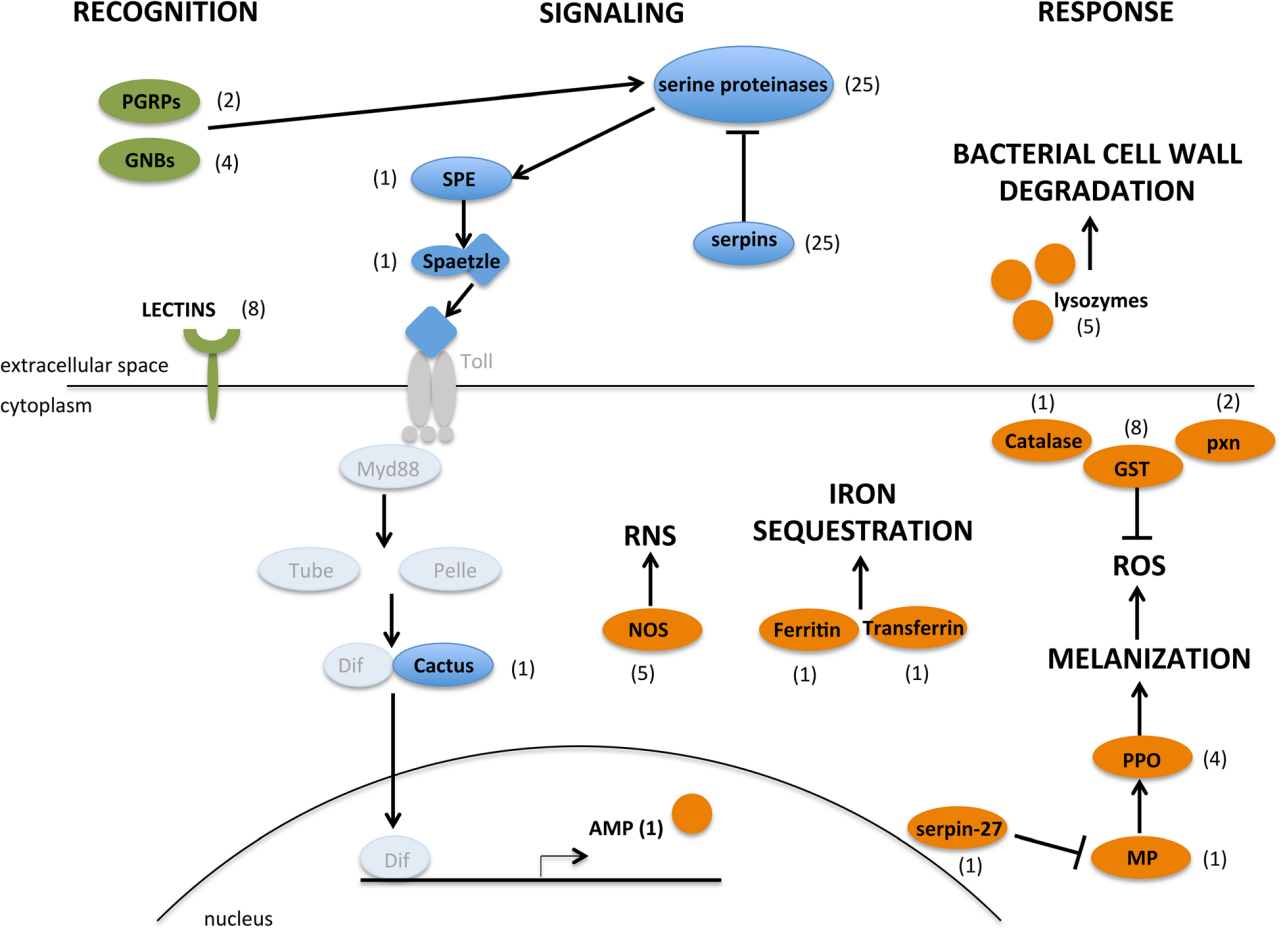
Schematic representation of key immunity-related genes expressed in the highly secreting pleuropodia of *S. gregaria*. Proteins whose transcripts were found in the pleuropodia are in black, number in the brackets is the number of upregulated transcripts. Proteins whose transcripts were not upregulated are in grey. Out of the total 25 serine proteases and 25 serpins, 14 and 15 are known to function in Toll signaling, respectively. AMP, antimicrobial peptide; GNBP, gram-negative bacteria-binding protein; GST, glutathione S-transferase; MP, melanization protease; NOS, nitric oxide synthase; PGRP, peptidoglycan recognition protein; PPO, pro-phenoloxidase; pxn, peroxiredoxin; RNS, reactive nitrogen species; ROS, reactive oxygen species; SPE, Spaetzle-processing enzyme

An observation that was not anticipated was the upregulation of genes for proteins involved in immunity [71, 72] (Figs. 6 and 9, Additional file 2: Table S13). This is especially interesting, because immunity related proteins have been found in the MF [55]. This is in agreement with that the cells in the pleuropodia are a type of barrier epithelium [71–73], which enables the contact between the organism and its environment. Barrier epithelia (e.g., the gut, Malpighian tubules or tracheae) constitutively express genes for immune defense.

**Fig. 10:**
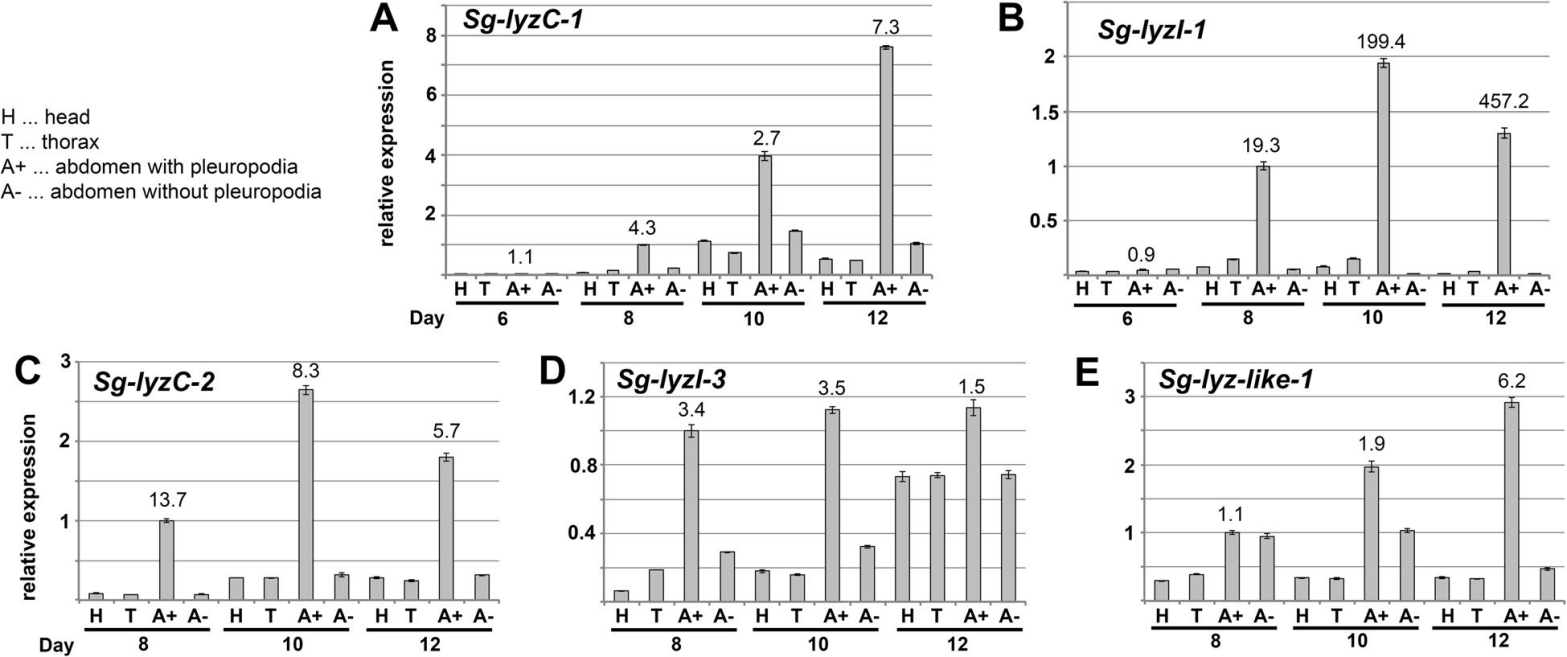
Real-time RT-PCR expression analysis of genes for lysozymes on cDNA from parts of *S. gregaria* embryos. cDNA was prepared from mRNAs isolated from parts of embryos at the age of 6, 8, 10 and 12 days. Labeling of the body parts is explained on the left of this Figure; see also an immage summary in Fig. 8a. For each age the same number of body parts was used (5–10) and RNA was resuspended in the same volume of water. Analysis of 3–4 technical replicates is shown. Expression in A+ 8 (abdomen with pleuropodia at stage when the organs first become differentiated) was set as 1. Numbers above A+ expression is fold change from A- of the same age

In total we found upregulated 99 transcripts (13 % of the upregulated genes) for immunity-related proteins. These include proteins at all three levels, the pathogen recognition, signaling and response (Fig. 9, Additional file 2: Table S13). From the four signaling pathways, Toll was upregulated, but not IMD or JAK/STAT, and from the JNK signaling we found c-Jun. Genes for a range of immune responses were upregulated, including production of reactive nitrogen species (RNS), melanization, genes for lysozymes and one antimicrobial peptide (AMP) similar to Diptericin.

The transcripts for lysozymes were among the most highly expressed (Table 1) and we chose to focus on them. Lysozymes are secreted proteins that kill bacteria by breaking down their cell wall. Our *S. gregaria* transcriptome contains nine genes for lysozymes, seven of which were upregulated (Table 4, Additional file 2: Table S14). The second most highly expressed DEG was a transcript for a C-type lysozyme *(Sg-LyzC-1)* that was previously shown to have anti-bacterial properties in *S. gregaria* [74] (Table 1). We examined expression of five selected genes on cut embryos by real-time RT-PCR (Fig. 10). Our data showed that the pleuropodia are the major source of mRNAs for these genes.

**Table 4:** RNA-seq differential gene expression of *S. gregaria* lysozymes in the highly secreting pleuropodia

Lysozyme type	Gene	UP/DOWN^a^	Fold change	Expression^b^
C-type lysozyme	***Sg-LyzC-1***	**UP**	**336.64**	**2 (0.26 %)**
	***Sg-LyzC-2***	**UP**	**134.71**	**37 (4.74 %)**
I-type lysozyme	***Sg-LyzI-1***	**UP**	**550.26**	**7 (0.90 %)**
	*Sg-LyzI-2*	ns^c^		
	***Sg-LyzI-3***	**UP**	**30.62**	**76 (9.73 %)**
	*Sg-LyzI-4*	DOWN	−34.41	1251 (81.50 %)
	*Sg-LyzI-5*	ns		
Lysozyme-like	***Sg-Lyz-like-1***	**UP**	**192.68**	**150 (19.21 %)**
	*Sg-Lyz-like-2*	ns		

^a^ upregulated (UP)/ downregulated (DOWN)

^b^ the DEGs (781 upregulated) were ranked according to their RPKM in descending order, the number (percentage) describes the position of the DEG in the ranked table; transcripts in bold were among the top 25 % most abundant

^c^ not significant

### Pleuropodia do not upregulate the pathway for ecdysone biosynthesis

Previous work has suggested that pleuropodia may be embryonic organs producing the moulting hormone ecdysone [43]. During post-embryonic stages, ecdysone is synthesized in the prothoracic glands and several other tissues by a common set of enzymes [75–77], some of which have been characterized in the locusts [78–81]. As shown in *Drosophila*, these genes are expressed in diverse cell types in embryos, and when the larval prothoracic glands are formed their expression co-localizes there [82–86].

Out of the nine genes critical for ecdysone biosynthesis, only one (*dib*) was upregulated in the highly secreting pleuropodia (Table 5, Additional file 2: S15). One gene (*spo*) was downregulated. The pleuropodia are not enriched in the whole pathway at any time of development, including around katatrepsis, in which experiments supporting the synthesis of moulting hormone were carried out (Additional file 2: Table S9, S16). Under the GO term “hormone biosynthetic process” enriched in the highly secreting pleuropodia (Additional file 2: Table S7, S17) we found a gene *Npc2*a that also functions in ecdysone biosynthesis in *Drosophila* [87]. It encodes a transporter of sterols, which include precursors of ecdysone. The enzyme functions as a general regulator of sterol homeostasis in diverse tissues and may have other roles in the pleuropodia than ecdysone biosynthesis. We conclude that our transcriptomic data provide little evidence that the pleuropodia are involved in ecdysone biosynthesis.

**Table 5:** RNA-seq differential gene expression of *S. gregaria* ecdysone biosynthesis enzymes in the highly secreting pleuropodia

Gene	UP/DOWN^a^	Fold change	Expression^b^
*shade (shd), Cyp314A1*	ns^c^		
*shadow (sad), Cyp315A1*	ns		
*disembodied (dib), Cyp302A1*	UP	5.71	431 (55 %)
*phantom (phm), Cyp306A1*	ns		
*shroud (sro)*	ns		
*spook (spo), Cyp307A1*	DOWN	−12.32	1368 (89 %)
*spook-like*	ns		
*neverland (nvd)*	ns		
*Cyp6t3*	not found		
*Cyp6u1*	na^d^		
*Cyp303a1*	ns		

^a^ upregulated (UP)/ downregulated (DOWN)

^b^ the DEGs (781 upregulated and 1535 downregulated) were ranked according to their RPKM in descending order, the number (percentage) describes the position of the DEG in the ranked table

^c^ not significant

^d^ not applicable (expression low to undetectable in both samples, transcript filtered out)

## Discussion

### Pleuropodia of *S. gregaria* release secretion granules from shortly before dorsal closure until hatching

Using TEM we showed that the glandular epithelium in the pleuropodia of *S. gregaria* fully develops shortly before dorsal closure, on day 8 in our staging (55 % DT), when granular secretion outside the cells becomes visible. On day 6 and 7, which surround katatrepsis (45 % DT), the glandular cells only begin to differentiate and do not secrete. This would explain why no digestive effect on SC was detected by Novak and Zambre [43] when using a homogenate from *S. gregaria* pleuropodia isolated at around katatrepsis.

Pleuropodia at stages before and after katatrepsis were previously examined by TEM in other orthopterans, *Eyprepocnemis plorans*, *Ailopus thalassinus* and *Ailopus strepens* [39]. Unlike our observations on *S. gregaria*, this study detected release of secretion granules already in stages preceding katatrepsis. Their images (presence of secretion granules outside cells that have long microvilli, thick layer of fibres released from the tips of microvilli forming the EC1 cuticle) corresponds to developmentally more advanced pleuropodia, of *S. gregaria*, around day 8 or 8–9 (compare e.g., Fig. 3a in [39], before katatrepsis with our Fig. 3h,i, before and just after katatrepsis). This might be because in different species the glandular cells differentiate at different speed or the samples studied by Viscuso and Sottile [39] were taken from embryos that were slightly older than described. The fine staging and sampling at precise developmental age in our study on *S. gregaria* supports that pleuropodia of orthopterans (Orthoptera: Caelifera) are not yet functional at katatrepsis. All pleuropodia that we examined from several 6- and 7-day embryos (before and after katatrepsis) gave similar results per stage (see Methods). Intense secretion from pleuropodia of *S. gregaria* that we observed after dorsal closure and close to hatching is in agreement with other TEM studies on orthopterans *E. plorans*, *A. thalassinus*, *A. strepens* [39] and *Locusta migratoria* [37].

### Pleuropodia of *S. gregaria* express genes for the “hatching enzyme”

Our RNA-seq analysis revealed that the secreting pleuropodia highly express genes encoding enzymes that are capable of digesting a typical chitin-protein insect cuticle. These include genes for proteolytic enzymes similar to those present in the MF and cuticular chitin-degrading NAGs and Chitinase 5. The pleuropodia also express genes for Chitinase 2 and Idgf, which have low effect on cuticular chitin digestion, but were shown to organize proteins and chitin fibres during cuticle deposition and for Idgf to have an immune function [63, 64]. These CHTs may organize the fibres in the cuticle secreted by the pleuropodia (Fig. 3).

We showed that, while the expression of the *Sg-nag1* and *Sg-nag2* started to rise in parallel with the differentiation of the glandular cells, the *Sg-cht5–1* and *Sg-cht5–2* transcripts raised shortly before hatching. Chitinase 5 is a critical chitin-degrading chitinase in insects: it is highly abundant in the moulting fluid and its silencing in diverse insects including the locust *L. migratoria* leads to failure in larval moulting [55, 62, 64, 67, 88] and as shown in *L. migratoria* also in moulting of the embryonic cuticle [89]. Our data indicate that the sudden rise in the expression of *Sg-cht5* in the pleuropodia at the end of embryogenesis and presumably secretion of this CHT into the extraembryonic space is an important component of the “hatching enzyme” effect [30, 31] in locusts and grasshoppers. Since silencing of this single gene in embryos of *L. migratoria* did not prevent hatching [89], we conclude that the other chitin and protein degrading enzymes produced in the pleuropodia, and perhaps the serosa (see below), are essential as well.

### Pleuropodia in some other insects could secrete the “hatching enzyme” and their function may also vary among species

There is evidence to suggest that the process occurs similarly in some insects. As in orthopterans, the pleuropodia of the rhagophthalmid beetle *Rhagophthalmus ohbai* release secretion after katatrepsis and SC rapidly degrades just shortly before hatching [17]. In the large water true bugs from the family Belostomatidae, the male carries a batch of eggs on his back. It is believed that the detachment of the eggs just before hatching is also caused by the secretion from the pleuropodia [90].

The molecular mechanism of SC degradation may also vary between insects and as previously hypothesized [43] the serosa may contribute to the SC degradation. The serosa of the beetle *Tribolium castaneum*, expresses *cht10* and *cht7* [33], of which the former CHT is important for cuticular chitin digestion. Silencing of *cht10*, but not *cht5* prevented the beetle larvae from hatching [62]. Transcripts for *cht10* were not upregulated in the pleuropodia of *S. gregaria*. This suggests that the SC is degraded by enzymes produced by both, the serosa and the pleuropodia and that the indispensable roles in cuticle digestion are played by different enzymes in different insects. Likely, the serosa, before it degenerates in mid-embryogenesis, releases some of the SC degrading enzymes, but these do not make up the complete cocktail that would be able to digest the cuticle efficiently. The secretion from the pleuropodia than adds the missing enzymes.

In some insects the pleuropodia may not be involved in hatching but have another function. In the viviparous cockroach *Diploptera punctata* [38], the secretion from the pleuropodia is very low and the large pleuropodia of the melolonthid beetle *Rhizotrogus majalis* have not been observed to produce any secretion granules at all [20]. In dragonflies, one of the more basal lineages of insects, the secretion likely has a different function than in orthopterans, because their SC is not digested before hatching [8]. The special epithelium in the pleuropodia shares features with transporting epithelia [36, 38] that function in water transport and ion balance [91]. Our data do not exclude this function, but it is yet to be tested. Our data do not support that the pleuropodia specialize in producing the moulting hormone [43] because they do not upregulate the whole set of the moulting hormone ecdysone biosynthetic enzymes. However, it cannot be excluded, that the pleuropodia produce some ecdysone intermediates; particularly products of biosynthetic steps catalyzed by the dib and spo enzymes that we found upregulated at some stages.

### The pleuropodia of *S. gregaria* are enriched in transcripts for enzymes functioning in immunity

We found that many of the genes expressed in the pleuropodia encode proteins involved in immunity [72]. This indicates that the pleuropodia are also organs of epithelial immunity, similar to other barrier epithelia in postembryonic stages (such as the gut) [73], which are in a constant contact with microorganisms. The pleuropodia differ from such tissues in that they are not directly exposed to the environment, but enclosed in the eggshell, seemingly limiting their contact with microorganisms. Proteins associated with immune defense are also found in the MF [55], where they prevent invasion of pathogens through a “naked” epidermis after the separation of the old cuticle from the epidermis in the process of apolysis. As found in the beetle *T. castaneum*, during the early embryonic stages the frontier epithelium providing the egg with an immune defense is the extraembryonic serosa [92]. The serosa starts to degenerate after katatrepsis and disappears at dorsal closure [44]. The pleuropodia of *S. gregaria* differentiate just before dorsal closure, suggesting that they take over this defense function in late embryogenesis. It will be interesting to clarify in the upcoming research whether apart from their role in hatching the pleuropodia are important organs for fighting against potential pathogens that have gained access to the space between the embryo and the eggshell.

### Evolution of the pleuropodia

Although only the functions of pleuropodia in digestion of SC and production of the moulting hormone in orthopterans were supported experimentally, current little data indicate that the function of these organs has been changing during evolution. In the diverse insect lineages that have them, the ultrastructure of the cells in pleuropodia appear similar, but the organs have different shapes. Hypothetically then, the glandular and/or water and ion transporting cells [20, 25, 35–39] were adapted to suit the needs of the developing embryo, such as digestion of the tough SC in orthopterans [30] or exchanging fluids and ions by particularly elongated pleuropodia in viviparous cockroaches [38]. In advanced insect lineages this type of cells is likely not needed and the pleuropodia do not develop. Future research by modern techniques is needed to uncover the diverse functions of these ancient insect organs and their role in insect evolution, physiology and development.

## Conclusions

Transcriptomic profiling of pleuropodia from *S. gregaria* supports that the conclusions that Eleanor Slifer drew from her experiments over 80 years ago that these organs secrete cuticle degrading enzymes, were correct. The pleuropodia likely have other functions, such as in immune defense. The pleuropodia appear to be true live embryonic organs and likely an important but neglected part of insect physiology. The sequencing data that we generated will in future studies enable to dissect the functions of these enigmatic organs in a detail.

## Methods

### Insects

*Schistocerca gregaria* (gregarious phase) originated from a long-term colony at the Department of Zoology, University of Cambridge. Eggs were collected into pots with damp sand in two- or four-hour intervals. The pots with eggs were stored in an incubator at 30 °C, in constant darkness and the sand was kept moist.

### Imaging of embryonic stages

Embryos and appendages were dissected in phosphate buffer saline (PBS). Whole eggs were treated with 50 % household bleach to dissolve the chorion. All were photographed using the Leica M125 stereomicroscope equipped with DFC495 camera and associated software. Photos were processed using Adobe Photoshop CC 2017.1.1. Photos of eggs and embryos (Fig. 2a and Additional file 1: Figure S1) had the background cleaned using the software.

### Immunohistochemistry on paraffin sections

Embryos were dissected in PBS and pieces including A1 were fixed in PEMFA (4 % formaldehyde in PEM buffer: 100 mM PIPES, 2.0 mM EGTA, 1.0 mM MgSO_4_) at room temperature (RT) for 15–30 min, washed in PBT (PBS with 0.1 % Triton-X 100) and stored in ethanol at − 20 °C. Samples were cleared 3 × 10 min in Histosol (National Diagnostics) at RT, infiltrated with paraffin at 60 °C for 2–3 days and hardened in moulds at RT. Sections 6–8 μm thick were prepared on a Leica RM2125RTF microtome. Slides with sections were washed with Histosol, ethanol and re-hydrated to PBT. Slides were placed in a humidified chamber, blocked with 10 % sheep serum (Sigma-Aldrich) in PBT for 30 min at RT and incubated with Phospho-Histone H3 antibody (Invitrogen) diluted 1:130 at 4 °C overnight, Alexa Fluor 568 anti-rabbit secondary antibody (Invitrogen) diluted 1:300 at RT for 2 h and DAPI (Invitrogen) diluted 1:1000. Sections were imaged with a Leica TCS SP5 confocal microscope and photos processed using Fiji (https://fiji.sc).

### Electron microscopy

For TEM embryos were dissected in PBS and pieces including A1 were fixed in 2.5–3.0 % glutaraldehyde in 0.1 M phosphate buffer (PB) pH 7.2 for a few hours at RT and at 4 °C for several days. Pleuropodia and legs were separated from other tissues and embedded into 2 % agar as previously [47]. Appendages in agar were incubated in solution of 3 % OsFeCN in cacodylate buffer with 4 mM CaCl_2_ for 1–2 days at 4 °C, 0.01 mg/ml thiocarbohydrazide (Sigma-Aldrich) for 20–30 min at RT in dark, 2 % OsO_4_ 30–45 min at RT and 1 % uranyl acetate (pH 5.5) at 4 °C overnight. Washing steps were done with deionized water. Samples were dehydrated in ethanol, washed with dry acetone, dry acetonitrile, infiltrated with Quetol 651 resin (Agar Scientific) for 4–6 days and hardened in moulds at 60 °C for 2–3 days. Ultrathin sections were examined in the Tecnai G280 microscope. From each stage at least three pleuropodia and three legs were examined; all replications showed similar morphology.

For SEM whole embryos were fixed in 3 % glutaraldehyde in PB, post-fixed with OsO_4_, dehydrated in ethanol, critical point dried, gold coated, and observed in a FEI/Philips XL30 FEGSEM microscope. Photos from TEM and SEM were processed using Adobe Photoshop CC 2017.1.1.

### Preparation of the reference transcriptome

The reference transcriptome includes transcripts that were assembled from (a) RNAs isolated from whole eggs and (b) RNAs isolated from legs and pleuropodia at a stage shortly before dorsal closure (sample “day 8–9”). (a) Whole egg transcriptome: Eggs from each 24-h egg collections incubated for the desired time were briefly washed with 50 % household bleach, washed with water and frozen in liquid nitrogen. Total RNA was isolated using TRIzol (Invitrogen), treated with TURBO DNase (Invitrogen) and purified on a column supplied with the RNAeasy Kit (Quiagen). The purified RNA from each of the 14 one-day samples was pooled into four: day 1–4, 5–7, 8–10 and 11–14. Ten μg of RNA from each was sent to BGI (Hong Kong). The total RNA was enriched in mRNA using the oligo (dT) magnetic beads and cDNA library was prepared using a standard protocol. 100 bp paired-end (PE) reads were sequenced on Illumina HiSeq 2000; the numbers of reads are in Additional file 2: Table S2. Non-clean reads were filtered using filter_fq. Transcripts from the samples were assembled separately using Trinity (release 20,130,225) [93]; parameters: --seqType fq --min_contig_length 100; −-min_glue 4 --group_pairs_distance 250; −-path_reinforcement_distance 95 --min_kmer_cov 4. The four assemblies were merged together to form a single set of non-redundant transcripts using TGICL software (version 2.1) [94]; parameters: -l 40 -c 10 -v 20. (b) Legs and pleuropodia transcriptome (age about 8.5–8.75 days): Appendages were dissected in RNase-free PBS and total RNA was isolated and cleaned as above. Ten μg of RNA from each leg and pleuropodium samples were transported to the Eastern Sequence and Informatics Hub, Cambridge (UK). cDNA libraries were prepared including mRNA enrichment. 75 bp PE reads were sequenced on Illumina GAIIX; the numbers of reads are in Additional file 2: Table S2. Reads were trimmed to the longest contiguous read segment for which the Phred quality score at each base Q > 13 (or 0.05 probability of error) using DynamicTrim (version 1.7) from the SolexQA package [95] and filtered to remove sequence adapter using Cutadapt (version 0.9) (http://code.google.com/p/cutadapt/). Sequences < 40 bp were discarded. The transcriptome was assembled using combination of Velvet (version 1.1.07) [96] (parameters: -shortPaired –fastq; −short2 –fastq; −read_trkg yes) and Oases (version 0.2.01) [97] (parameterss: -ins_length 350): the contigs that were output by Velvet were used by Oases to build likely transcripts from the RNA-seq dataset. K-mer sizes of 21, 25 and 31 were attempted for the two separate samples as well as the combined samples and optimal K-mer sizes of 21 were found for both samples.

Transcripts from the egg and legs plus pleuropodia transcriptomes were first merged with evigene (version 2013.03.11) using default parameters. Because this selection of transcripts (Selection 1) eliminated some genes (represented by zero transcripts, although the transcripts were present in the original transcriptomes), we repeated the step with less strict parameters (cd-hit-est - version 4.6, with -c 0.80 -n 5). This Selection 2 contained several genes represented by multiple transcripts, therefore we aligned Selection 1 and 2 to each other. Selection 1 was then completed by adding the missing transcripts from Selection 2. The resulting selection was edited as follows. Several redundant transcripts were removed manually: these were found by blasting diverse insect sequences against the transcriptome using the local ViroBLAST interface [98]. Some transcripts were edited manually: e.g., when we found that two transcripts were combined into one (*S. gregaria* transcript blasted against sequences in GenBank resulted in different parts in high scoring alignments against different protein sequences) we split the transcripts, or when the transcript had a frameshift mutation that was not in the other transcripts from the mRNA, we corrected this. These manually changed transcripts were found during a random inspection of the selection. The resulting selection was blasted against itself (Blast+ suite, version 2.6.0) and if there was an alignment spanning ≥300 bp with a sequence identity of ≥98 % the redundant shorter transcript was removed. Transcripts < 200 bp were discarded. These steps were carried out in R [99] using the Biostrings package [100].

### Sequence analysis

Basic transcript analysis was done using CLC Sequence Viewer7 (QIAGEN). Signal peptide and transmembrane regions were predicted by Phobius [101]. Conserved domains were identified using SMART (http://smart.embl-heidelberg.de/). The reference transcriptome was annotated using Trinotate (version 3.1.1) [102]. The longest candidate ORF of each transcript was identified using the inbuilt software TransDecoder [102]. The transcriptome was blasted against Uniprot sequences of *Schistocerca gregaria, Locusta migratoria, Apis melifera, Tribolium castaneum, Bombyx mori* and *Drosophila melanogaster* (blastx with default parameters and -max_target_seqs 1).

### RNA-seq expression analysis

Pleuropodia and legs from embryos at the same age (day 4, 5, 6, 7, 8, 10, 11, 12 and 13) were dissected in RNase-free PBS and total RNA was isolated as described above, but cleaned with RNA Clean & Concentrator (Zymo Research). One μg of RNA from each sample was sent to BGI (Hong Kong). cDNA was prepared including mRNA enrichment as above. Over 45 millions of 50 bp single-end (SE) reads were sequenced from each sample (Additional file 2: Table S2) on Illumina HiSeq 2000. A pair of samples from embryos that was used for the preparation of the reference transcriptome, part (b) above (“day 8–9”), was included in the expression analysis, but prior to mapping, the 75 bp PE reads were trimmed to 50 bp, using Trimmomatic in the paired-end mode (version 0.36) and using the CROP function (CROP:50). Each sample for expression analysis contained tens to hundreds of appendages. A single sample from each pleuropodia and legs was sequenced per stage.

The quality of the sequenced reads was assessed using the FastQC. All samples showed a Per base sequence quality > 30. Reads were mapped to the reference transcriptome using Bowtie2 (version 2.2.5) with default parameters and –local alignment mode [103]. Trimmed pairs of reads were concatenated for each stage and treated as single reads. A PCA plot was prepared using the plotPCA() function in the DESeq2 R package [104]; the count matrix was transformed with the rlog() function. The plot showed that differences in sequencing type and processing of SE and PE samples had no noticeable effect on the results. The R package HTSFilter [105] was used with default parameters to remove transcripts with constantly low expression; 12,988 transcripts were left.

The differential expression analysis was performed using the NOISeq R package (version 2.22.1 [106];). Reads were normalized using the RPKM method [107]. DEGs between legs and pleuropodia for each stage were searched using NOISeq-sim; parameters for simulation of “technical replicates” prior to differential expression analysis without replicates: k = NULL, norm = “n”, pnr =0.2, nss =5, v = 0.02, lc = 1, replicates = “no”. DEGs between highly secreting pleuropodia and equally aged legs (samples from day 10, 11 and 12 treated as replicates) were searched using the NOISeq-real algorithm; parameters: k = 0.5, norm = “n”, factor = “type”, nss = 0, lc = 1, replicates = “technical”. Thresholds for significant differential expression were probability (“prob”) > 0.7 for single stage comparisons and > 0.8 for the triplicated comparison, RPKM > 10 and fold change > 2 for all comparisons (thresholds were set arbitrarily based on the values for the genes whose expression dynamics in the pleuropodia are known, Additional file 2: Table S4).

### GO enrichment analysis

The transcriptome was blasted against the UniProt/Swiss-Prot database. GO enrichment with blast hits of an e-value ≤1e^− 5^ was performed using the R package GOSeq (version 1:30.0 [108];) implemented in the Trinotate pipeline (see above). Enriched GO terms were summarized and visualized using REVIGO [109]. Dot plots were prepared from DEGs having RPKM > 50, fold change > 3.

### Real-time RT-PCR

Tissues were dissected, total RNA was isolated and DNase treated as described for sequencing and cleaned with RNA Clean & Concentrator (Zymo Research). cDNA was prepared from 0.5 μg (legs, pleuropodia) or 1 μg (cut embryos) of the RNA using oligo-dT primer (Invitrogen) and ThermoScript RT-PCR System (Invitrogen) at 55 °C (lower amount of RNA from legs and pleuropodia, compared to the amount of RNA from whole cut embryos, was used because this RNA was in a short supply and difficult to obtain since these appendages are small and had to be dissected). PCR reactions (20 μl) contained 5 μl of cDNA diluted to 40 ng/μl, 10 μl of SYBR Green PCR Master Mix (Applied Biosystems) and 5 μl of a 1:1 mix of forward and reverse primers (each 20 nM in this mix). Reactions were run in the LightCycler480 (Roche) and analyzed using associated software (release 1.5.0 SP1) according to comparative Ct method and normalized to the *eEF1α* gene. Amplification was 40 cycles of 95 °C for 10 s, 60 °C for 15 s, 72 °C for 12 s. Primers (Additional file 2: Table S18) were designed using Primer3PLUS [110]. To check for the presence of a single PCR product, the melting curve was examined after each run and for each pair of primers at least two finished runs were visualized on a 2 % agarose gel.

## Supplementary information

**Additional file 1: Figure S1.** *S. gregaria* embryonic stages used in this study. **Figure S2.** External features of developing hind legs and pleuropodia. **Figure S3.** Cross-sections through developing hind legs and pleuropodia. **Figure S4.** Ultrastructure of epidermal cells in developing hind legs. **Figure S5.** Amino acid sequences and conserved domains of *S. gregaria* chitin degrading enzymes. **Figure S6.** Phylogenetic trees of chitin degrading enzymes in *S. gregaria* and other insects. **Figure S7.** Real-time RT-PCR expression analysis of *Sg-cht7–1* and *Sg-cht10–1* on cDNA from parts of *S. gregaria* embryos.

**Additional file 2: Table S1.** Embryonic transcriptome of *S. gregaria*: numbers of sequenced reads and assembled transcripts. **Table S2.** RNA-seq expression analysis: numbers of sequenced and mapped reads. **Table S3.** Number of differentially expressed genes at selected levels of stringency. **Table S4.** Differential expression of genes, whose expression dynamics in the early stages of pleuropodia development is known. **Table S5.** Comparison between differential expression of selected genes obtained by RNA-seq and real-time RT-PCR. **Table S6.** GOs enriched in the downregulated DEGs from the highly secreting pleuropodia (joined sample 10, 11 and 12 days). **Table S7.** GOs enriched in the upregulated DEGs from the highly secreting pleuropodia (joined sample 10, 11 and 12 days). **Table S8.** GOs enriched in the downregulated DEGs from each developmental stage . **Table S9.** GOs enriched in the upregulated DEGs from each developmental stage. **Table S10.** Genes for core transporters expressed in the pleuropodia. **Table S11.** *S. gregaria* genes for proteins with GO “proteolysis” that were upregulated in the highly secreting pleuropodia. **Table S12.** Differential gene expression of *S. gregaria* homologs (or close relatives) of known genes for MF proteases in the highly secreting pleuropodia. **Table S13.** Immunity-related proteins expressed in the highly secreting pleuropodia. **Table S14.** Genes for lysozymes identified in the *S. gregaria* embryonic transcriptome. **Table S15.** Genes for ecdysone biosynthesis enzymes identified in the *S. gregaria* embryonic transcriptome. **Table S16.** RNA-seq differential gene expression of *S. gregaria* ecdysone biosynthesis enzymes in the pleuropodia at diverse stages. **Table S17.** *S. gregaria* genes with GO terms “hormone biosynthetic process” upregulated in the highly secreting pleuropodia. **Table S18.** Sequences of primers.

## Data Availability

The datasets generated and analysed during the study are available in the NCBI repository: BioProject ID PRJNA524786 (the reference transcriptome described in this paper is the first version, GHHP01000000) and GSE128394.

## Abbreviations

A1: First abdominal segment
CHT: Chitinase
DEG: Differentially expressed gene
DT: Developmental time
EC1, EC2, EC3: The first, the second, the third embryonic cuticle, respectively
GO: Gene ontology
LEG: Hind leg(s)
MF: Moulting fluid
NAG: β-N-acetyl-hexosaminidase
PCA: Principal component analysis
PLP: Pleuropodium (pleuropodia)
RPKM: Reads per kilobase of transcript per million reads mapped
SC: Serosal cuticle
SEM: Scanning electron microscopy
T3: Third thoracic segment
TEM: Transmission electron microscopy
