# Supplementary material for: Transcriptome of pleuropodia from locust embryos supports that these organs produce enzymes enabling the larva to hatch
Source: Front Zool. 2020 Jan 16;17:4. doi: 10.1186/s12983-019-0349-2 (PMC6966819; doi:10.1186/s12983-019-0349-2)
Supplement: Supplementary file 1 — Additional file 1: Figure S1. S. gregaria embryonic stages used in this study. Figure S2. External features of developing hind legs and pleuropodia. Figure S3. Cross-sections through developing hind legs and pleuropodia. Figure S4. Ultrastructure of epidermal cells in developing hind legs. Figure S5. Amino acid sequences and conserved domains of S. gregaria chitin degrading enzymes. Figure S6. Phylogenetic trees of chitin degrading enzymes in S. gregaria and other insects. Figure S7. Real-time RT-PCR expression analysis of Sg-cht7–1 and Sg-cht10–1 on cDNA from parts of S. gregaria embryos. [file 12983_2019_349_MOESM1_ESM.pdf]

FIGURE S1

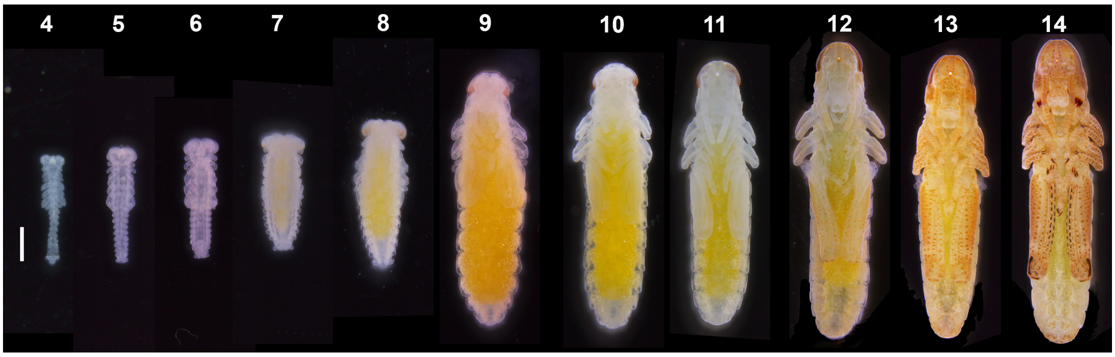

Figure S1. ***S. gregaria*** **embryonic stages used in this study.**

Images of live embryos dissected out of the eggs; imaged under a stereomicroscope. Eggs and embryos of *S. gregaria* typically slightly vary in size. Numbers indicate age in days. Scale bar: 1 mm. Background in photos was cleaned (see Methods).

FIGURE S2

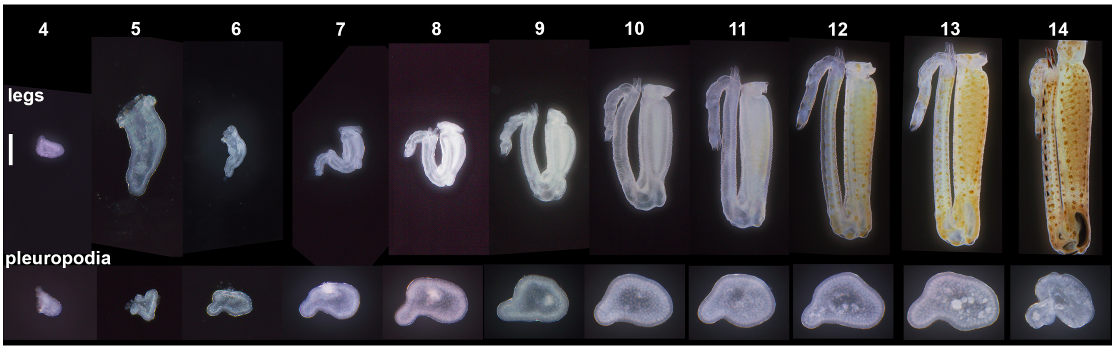

Figure S2. External features of developing hind legs and pleuropodia.

Compare the sizes of the appendages; imaged under a stereomicroscope. Numbers indicate age in days. Scale bar: 0.2 mm for all pleuropodia and for legs at days 4 and 5; 0.5 mm for legs at days 6-14.

FIGURE S3

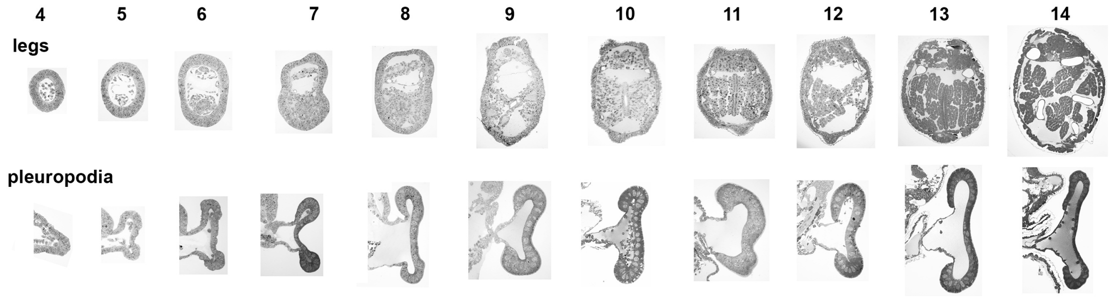

Figure S3. Cross-sections through developing hind legs and pleuropodia. Toluidine blue stained semithin sections of appendages embedded in epoxy resin. Numbers indicate age in days.

FIGURE S4

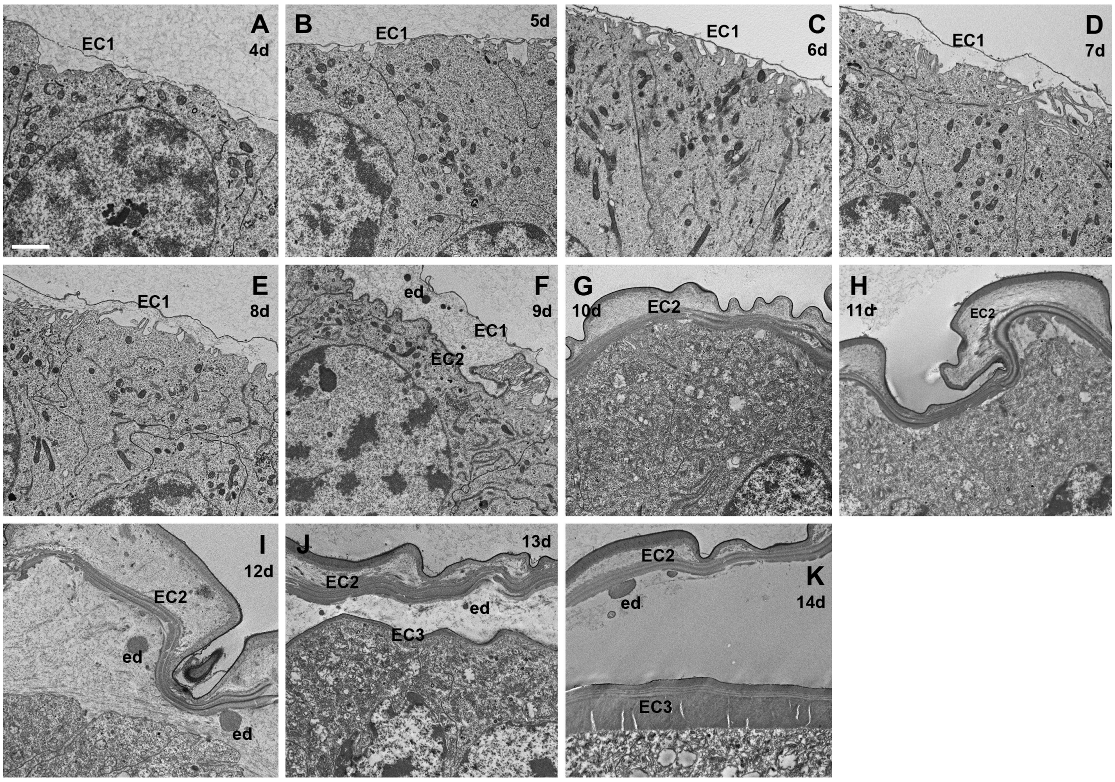

Figure S4. Ultrastructure of epidermal cells in developing hind legs. TEM micrographs. Compare with pleuropodia in Figure 3. Note the three different cuticles and appearance of ecdysial droplets (ed) during embryonic

moulting. EC1, EC2, EC3, the first, the second and the third embryonic cuticle, respectively (EC3 becomes the cuticle of the first instar larva). Scale bar: 2 µm.

FIGURE S5

(A)

Sg-nag1
MSVISTTVLVFALYGIFSCFATQAEEERPVWTWECRESRCEKVAAGEGEAQSLGACRLSCDPWATLWPRPRGGLQRTPGRLLA
LNPYSVSVEAAGRDLQPGVRQLLQEAGRIFHRKVERKARTGAKLRSAGERRSLFVTLTVSDGQTRSFHTDTSEAYSLSISEVTAG
RVNAAVTADTFFGARHALETLYQLIVYDDINKQLLLLSEINLSDSPAFPHRAIALDTARSYFSVASIKRTIDAMAANKLNTFHW
HITDSHSFPFVSETFPKLSQYGAYSPEKVYTPDEIKSVVEYARVRGVRIIPEFDAPAHVGEGWQWVGDNATVCFKADPWSQYCV
EPPCGQLNPTSEKMYQVLAGIYKDMLNVFDSDVFHMGGDEVNMNCWNTSEVITDWMDANGIPRTEEGLHELWDRFQSRAY
SLLAEANGKKELPVILWTSTLTDVAHVDKYLDNKRYIIQIWTRGTDLVIPELIRKGFRVIFSNYDALYFDCGFGAWIGSGNNWCS
PYIGWQKVYDNNVWDLLSAFGIDVGEGSEARKLVLGSEAALWSEQADEFALDGRLWPRAAALAERLWTDPVEGWMSAEHRF
LIQRQRLVDEGIAADTIEPEWCLQNQGHCYA*

Sg-nag2
MAPAPPAPHLLALTLLLTLLPSPPVVWANSPRWQWTCDSGLCVRSEAPPEPRLDAELEETVVQRSVHRLRPPWPSHELCRLT
CGPYGALWPRPTGHTLIADALVPFNPATARFDLSAVAGEQGRELVDAASRRWVRDLQHALAASGGHGGGEVAGAAAGAGT
DVLVTVLTRDSPQALSWETDETYTLDVASSGHEVRVTVSAQTVWGALHGLTSLRQLVGCCSEDGAALMVAEARIVDGPVYAH
RGLLLDTARNFLPVETMMATMDAMAASKLNVLHWHATDSQSFPLLLPRVPQLARWGAFSARETYSSQQVSALLGYAHARGI
RLLLELDAPAHSGQGWQWGEAEGLGALALCVGQQPWRRLCIQPPCGQLNPANPRLVGVLADVYRDVVDLWPPGQPLHMGG
DEVSYSCWNSSAEVLEYMSKRRWDRSQDGFLRLWAEFQQAALEALDAARGSSDVPAILWSSHLTRPGNIERFLNSSRYVIETW
VEGGDPLPQQLLALGYRLVVATKDAWYLDHGFWGSTRYHDWKAVYSNRLPGSMAQGVLGGEVASWGELVDDQSLDARLWP
RAAALAERLWSNPGASAREAEPRLHAHRARLVAAGVRPEALAPRYCVLNEGACQ*

Sg-fdl
MSRQRLLWRLLGAALALTVAGLAAPPLFRLLVSPHSAANSVAGRRVYSSDPGPWTWSCESGRCVRALWQGGTQVSLDTCQW
TCAGWEAPLWPRPTGALRLANSTAALPEDLDVRLRLSGPQHEDTRGLLAAATERLARHLQLVRPAWAGRVACDAARGATVA
RLTVFVKLDADGSRPTGQLTLDTDESYRLQVRRESQDLQAEIDARSFFGARHALETLSQLAWWDPVSGCVHILDSAIVKDAPKF
RHRGLMVDTARNFIPLEALQRTVDAMASNKLNTLHWHLTDSTSFPYLSRALPTMARYGAYSPEQVYSMEDVSRLAEFARERG
VRLVVELDVPAHAAAGWPTEQVSCSEQRGSAANAPLVQQQQHRQNEDNGLQYRQEERRERRAQHGGEQQPAWWELCGQPP
CGQLPPADEAAFGTLRTLYQELRQASGASDVAHLGGDEVSAECWGGVRGERLWSLWGGFMRRAHRELVAASQGNPPTAVLV
WSSELTAPHNLRRYFDPSTHVVQVWGGSKWNETLPVLLAGFRAVVSHVDAWYLDCGWGDFRSGGPGPCGPVATWQTVYSH
RPWAAFPPGARSRLLGGEACLWSEKVDDQTLDVRLWPRAAALAERLWSDPPAGVHPDLPPPGSPQRDEPTLRRAYQRLSHH
RERLVARGVRAEAMWPRYCHLNPGACF*

Sg-hex
MGKKVEVVLCACVCVGLLLTVTAAEPLPRYITEPGPTVKATQGAVWPKPQNEQRFGGSVLIVPGNFTFQVEGPECDILSEAVSR
YEAILKEEAAIKGPRNASEASTQLSALLVRLDGECGDRPVFGMDESYELRINSPDLPGAMLLTSASVWGILRGLETFSQVATRVK
TADALILDNLAIADIPRFSHRGLLLDTSRHFIPVSYIKKTLDAMAYNKMNVFHWHIVDDQSFPYQSAAFPLLSEKGSYDPERFVY
SPADVAEVIEYARVRGIRVVPEFDTPGHTRSWGEAYPDLLTPCYNATGSPDGTYGPIDPTKNFTYEFLQTLFEEIVNVFPDEYFH
LGGDEVGFECWESNQDILDFMSEHNITESKDLESYYIQKIVDIASNLNSKSIVWQEVFDNEVRLSADTVVHIWTGDRNEELDSV
TAAGHYTLLSQCYYLDRFRYFGGDWHKFYNCEPLDFSADNVYQYDLVIGGEAAMWSEFVDESNVESRVWPRASAVAERLWSP
MNVTDIDEAATRIEEHYCRLRRRGINAQPPNGPGYCV*

(B)

Sg-cht5-1
MRTSAAWFLAVAGLCVVFCPPLVSGNVGDRGRVVCYFSNWAIYRPGIGRYGIDDVPASMCTHLVYSFIGVSNVTWGVLVIDPE
NDVENHGFANFTALKSKYPGLKTQLAIGGWAEGGRKYSAMAAVPARRRSLIASVVEYMKRYGFDGFDLDWEYPGAADRGGSF
SDKNHFKCFVQELREAFDAEGQGWEITMAVPLAKFRLQEGYHVPELCELVDAIHVMSYDLRGNWAGFADTHSPLYKRPHDQ
WAYEKLNVHDGLKLWQDMGCPAHKLVVGVPFYGRSFTLSAGNKDYKLGTYINKEAGGGKPGNYTQAKGFLAYYEICLEIQEVG
GWTEKWDEAGKVPYAYKGTQWVGFENPKSVQIKMDFIKAKGYGGAMTWAIDMDDFRGVCGPKDALISVMYNNMKDYIVPD
IQYSTTKRPDWDRPPPCDGKKPGAAPASTTTRRPTAAPTQSTTRRPAPTTTAAPSSSSSTTTTRRTTTASRPSTQPPPPPAAPD
DNELPPAAIDCSDGDFVPHHDCSKYYRCVYGKPVEFSCYEGTVWNPQLRVCDRPNDVHRTDCSMAKLHS*

Sg-cht5-2
MRAATQVGLLLAVALALAAASDEDTTPLDSSTGSPTNSVDEESSSSENAAVLSGGQRRGRVTCYFESWAVYRKRLRYGIEDIPG
DMCTHIIYSFVGLNNVTWELQVLDEKLDVQDGGFENFTALRQEFPGVRLQVALGGWAEGGHNYSAMVGDPARRASLVRSAVA
FLHRYGFDGFDVDWEYPGNAPRGGVPEDKDDFLCFMQELRVAFDAEGLGWELTMAVPLTEDKLRDGFHVPQLCSIVDAVHV
MAYDLRGEWDHFADVHSPLYRRPHDTGAYAKINTHDGLLLWEQLGGSSWGCHSTATPTNCVPTSPTTLPVLASFRAPEMTSA
E*
(based on alignment with homologous sequences this transcript might be misassembled and the amino acid sequenced prematurely terminated by introduction of a stop codon)

Sg-cht10-1
MWRPVALSLWLLLATSRGLHVPPADEPSFVRDAVEAPPGQSLALRRSATASRPRLPAFGTRQLPLRQAVESPPMAARLRSSER
LPLRDAVEHVPYEALPGAPTASEAFSLWRGFGDWLPENLPSTRQFNHSFAWWHDAIIAKLSLGGPRTKPPSLQAPSTHTSGIR
QFKVVCFVEGWAGYRRDPMRFTTADIDPFACTHIIYAFAVMDPHDLHIKPQDEQYDIIQGGYRSIVGLKRQNPQLKVMISVGG
WPEERRKFAEMTASASTRREFIRSVLHFIDEYGFDGIDLDWEYPGAADMGGSAREKEHFSLLVEELAEAFAPRGSVLSASVSPS
RFRVEDGYDVPRLARRLDFLNLMAFDLLTEQDAAADHHAPLTQRKHDYGLAVFYNVDYAVRYWLRKGARRDQLVVGIPFHG
HSFTLQDEAKNSPGAPVKGLGKEGPYTQEKGFLAYFEILQLLEEGHWMKATDDVGSPYMVKGNQWIGYEDQRSIATKVMYIK

KNLLGGAMVWALDLDDFEGAYGQKWPLLSVVKKGLLETTPQSDQQQASQEPTHVTPPIAGVPVSVDSSQYNCSGRGYVRDSASCQIYHRCEWGMKHTYICPEGLHYDSRTQLCDWPQIANCPMDNSSQRIEQENQSEVACNEEGLMEDPKDCNRYYMCHKGVAQHYSCMLGQYFNVQKGICEYGSCMPKAPQDNIPSSQTRNLVGEDHYKVVCYYASWAWYRKEGGKFVPEHIDPTLCTHIVYAYASLDPNTLTMKYFDERADKENNFYERLTELPKKSGHHQQHASDVTVMIGLGGWTDSAGDKYSRLVSEGSARRRFVSKAVEFLHRHQFGGLHLDWDYPRCWQSNCGRGPTSDKPNFTKLVQELRQAFKKQNPPLALAISISGYHEVIDEAYDLAELGRNTDFMSVMTYDYHGSWEKSTGHVSPLYHRNGDIFPMYNTNDTMEYLVNKGAPRDKLLVGIPFYGQSYTLENPSNHDIGAPATGPGLAGEFTMQPGMLAYYEICDRVRNNFWKIGRDRFGATGPFAYAGNQWVSFEDTKSVKEKAKYIKNMGYGGAMTFTLDLDDFENRCCRGAFPLLRSINRVFGRIPDSAEPSGDDCTRPPPPVTPPPPTYTTGVDSGDHRPTTPISTTHQHPTSPKPSTTEYPWW

Sg-cht10-2

PSTTTSTTTSTTTTTTTTTTSTTTTPRPTTRPTTMSTTEYPWWTPSTTSTTRKPPTTRPTTTSTTEEYPWWTPPSTTKKPTTSSTTEQPWWTPSTTSTTSTAAPTTTMTTTEKPWWSTTPQKPLPPDSGPCEAGVYYPDPTNCNAYYRCVLGELRKEFCAGGLHWNPDKKVCDWPSESKCDTKEPSETTVGSTTSSTTENPWWTPSKPSETQATTTTTEVPWWSTTRPPRPPTTEGNSEWVTTSRPTTTQQPSEEVSECMNGQYYPVAGSCKSFYICVNGRLIKQTCAPGLVWNQDQTMCDWGFNVKCADDSEREAVHKAQPDDPCNQGALNPYPGDCTRYLYCQWGRYHEADCAAGLHWNEMEKICDWPENAKCTDMESGSEAPAASSQKPVTEMSTSWTTAAPTTKPPWTWATTTTVKPVTTTSTRAPPAQGPPISGYFKVVCYFTNWAWYRRGLGKYVPEDIDANLCTHIVYGFAVLDYENLIIKAHDSWADFDNKFYERVVAYKKKGLKVSLAIGGWNDSAGDKYSRLVNSPSARRRFIKHVLEFLEKYGFDGLDLDWEYPVCWQVDCAKGPASDKSSFAALVKELRQAFEPKGLLLSSAVSPSKTVIDAGYDVKTLAENLDWIAVMTYDFHGQWDKKTGHVAPLYFHPDDDFYFFNANFSINYWISEGAPRRKIVMGMPLYGQSFQLEKASTNGLNARSTGPGQAGEFTRAAGFLAYYEICDRIKNKGWTVVQDPERRMGPYAFKGNQWVSFDDVAMIQQKSEYIRKMGLGGGMIWALDLDDFRNRCGGGTHPLLNTIRTVLAAPPGGDGATEMPPSWSTPGGGQPTMSTEEWMSSTSISSTEITDSGHHSTQDSGGEVTSVSPAITTTNRPAHPGTSSSPPPPPSQGEFKVVCYFTNWAWYRQGVGKYLPNEIDPDLCTHIVYGFAVLNGDRLTIKPHDTWADYDNKFYEKVTEYKKKGIKVLVAIGGWNDSAGDKYSRLVNSPGARRRFIEDVIDFIEQNNFDGLDLDWEYPKCWQVDCKKGPDSDKEAFAAFVRELRAAFNPKGLLLTAAVSPSKAVVDAGYDVPTLSQNLDWIAVMTYDFHGQWDKITGHVAPMYTHPEDVDVTFNANFSIHYWIQKGASPKKIVMGMPMYGQSFSLADNSDHGLNAPTYGGGEAGESTRARGFLSYYEICTNIQKKGWRVVKDPEGRMGPYAYLRDQWVSFDDTSMIRYKSNFIRRMGLGGGMIWALDLDDFRNVCSCEKYPLLKTINRVLRGYPGPGPNCDIEATEKPGSEETDNRIHPTIPPTSSTNNWNVISGGGGIVPKDPTCGNRLFAPHDKDCNKYYLCQYGDFMEQSCPQGLYWNKDHCDWPSNTDCSKEDSSVINPAPIASTQEPEMSSTTENIHMSESTVTTSIRPSEPGTSTVMTPSGDYMVVCYFTNWAWYRQGLGKYLPSDIDTSLCTHIAYGFAVLDGNSLTIKPHDSWADLDNEFYTKVSGLKKKGIKVLLAIGGWNDSLGDKYSRLANNPSARRKFVEHVVKFIEKYGFEGLDLDWEYPKCWQVDCNAGPDSDKQGFADLVKELSMAFKPRGLLLSSAVSPSKVVIDSGYDVPVLSQYFDYISVMTYDFHGHWDKQTGHVAPLYYYPGDTYDYFNANFTMHYWIEKGADRKKLIMGMPMYGQSFSLADAKNHGLNAKSYGPGEAGEFTRAGGFMAYYEICYNVKSKGWTTVRDPEGRIGPYAYRGNQWVSYDDVSDIRRKTQFIKELGLGGGMIWALDLDDFRNRCGCGTYPLLRTINSELRGLTANTHDCT*

Sg-cht7-1

MIAPRCVWRAALWCVVIILLADLVYSASSTGRRRLRRPGGSSSSSTTSSSSSTSTKVRTRDQETSASVNRFRVRNRLTPPGANRKSGSGSAVAAASDKSGGYKVVCYYTNWSQYRTAHGKFLPEDITPDLCTHIIYAFGWLKKGKLTSFEGNDETKDGKVGLYERVMALKKANPKLKVLLALGGWSFGTQKFKAMSETRYTRQTFIYSAIPYLRKHDFDGLDMDWEYPKGTDDKKNFVLLLKELREAFEAEAQEVKQSRLLLSAAVPVGPDNVRGGYDVPAVASYLDFINLMAYDFHGKWERETGHNAPLYAPSSDSEWRKQLSVDHAATMWVKLGAPKEKLVIGMPTYGRTFTLSNPSNFKVNAPASGGGKAGDFTKEGGFLAYYEVCDMLKKGATYIWDDEMKVPYAVMGDQWVGFDDERSIRHKMKWLKEGGYGGAMVWTVDMDDFTGTVCGGGVKYPLIGAIREELRGVSRGPNAKDVDWSKVARTVSLEATTKPAPIKIDVSEVLNRVRKPTKQAPADLSNEVIDLNSRPAQVFCYMTSWSGKRPGAGKFSPEDVDPSLCTHVVFAFATLKDHKLAPANDKDDGLYERVIALREKNPQLKVLLAIGGWAFGSTPFKELTSNVFRMNQFVYDAIELLRDFKFDGLDVDWEYPRGADDRAAYVSLLKELRMAFEGEAKTAEQPRLLLSAAVPASFEAIAAGYDVPEISKYLDFINVMTYDFHGQWERQVGHNSPLYPLESATSYQKKLTVDFSAREWVKQGAPKEKLLIGMPTYGRSFTLVDTSKFDIGAPASGGGAAGRYTAEAGFMAYYEVCDFLHHDNTTLVWDNEQQVPFAYRGDQWVGFDDERSLKTKMGWLKELGFGGIMVWSVDMDDFRGQCGAGKYPLLTSMRQELRDYRVQLEYDGPYESRGPLGAYTTKDPTSVSCEEEDGHISYHPDKADCTMYYMCEGERKHHMPCPSNLVFNPNENVCDWPENVEGCMHHTQAPPAARRR*

Sg-cht7-2

MTWPPPPLLLSLLVLLATSASARFVSTHDVTPCAVEALAPSDKALLCYYEGRLSVYQLDPCLCTHIVFKDAAVVSDNFGLKIVSDVSGASLLRARSPSLRTVLGLRLSGAVARAALASPSRRLALARDAARRLYAHHLDGIELSVDDDEAASAAAADAAPAATARQGLVALLKALRTALDSHGREKRDYLVSEQVFDDFTTQEYEPTWSDGSSRKSRRRATTTTTTTSTTESPEETAARYLELERDAQNAQLLLSLPTKPETIAKRYDVKNITRYVDYVVLRTQAMTDDSERGLVYHPSRLMGLDDMLNADAVVDLVTSLGASPAQLVITLPGQATAFELRREDRTEPRSPASGAPRTISQPELCRALSRGNWTLERDEDQTAPYAYSGRRWIAFDDALSASIKGKYAVVRGLAGTAVDAADALDWQGTCGAPASQLRALHSALAQLRRSSRGALLHGLE

Sg-cht7-3

DKGMPKNKIIVGIPTYGHSFRLINAENHGWSAPASGYGKIGSKGFVSYPEVCQFLHSTGSKYIFDKNFEVPYAYQGLEWISYDDECSVMYKAKYIASSSYGGAMVFSLNVDDHQGVCAGTTFLLTTQIRNILGVSWQ*

Sg-cht2

MQQLAPLAFVLAFLAAAFAASPLGHNKAVVCYVSSWAVYRPGNGVFTVSDINPNICSHLVYAFAGLNATDNTIITLDKYNDLEEDYGKGNYKKITGLKNQYPHLKVSIAIGGWNEGSANYSHMASTPTTRQQFIRSVVNFLRKYNFDGLDLDWEYPTQRGGVPSDRENFVALVRELRQEFDKNGWLLTAALGASTAVIEKAYDVPMLGKYLDYMHIMCYDYHGTWDKMTGANAPLYGSSPSDTLSVDNSIRYYLKLGAPAKKLLMGVPLYGRTFMSDANANMGGLGAPAEEKSFQGPYTKEDGYMGYNEICLELKTNSSMWTIMWDDKSSTPYAVSTNKVIVYDNAKSLTEKVNLAMKLELGGIMVWPLDTDDFRGECSEGIYPLMHTINKAIVQSSQQKSDSSGMKVPDSTAAASCGCASLIFLSFLYLFQL*

Sg-cht6-1

VCYYTNWSVYRPGTAKFTPQNINPYLCTHLIYAFGGLSRENGLRPFDKYQDIEQGGYAKFTGLKTYNKDLKTMLAIGGWNEGSTRFSPLVADAERRKEFVKNVLRFLRQNHFDGLDLDWEYPAFRDGGKSRDRDNYALLVKELREEFDRESEKTGRPRLLLTMAVPAGIEYIDKGFDIASMNKHLDFMNILSYDYHSAFEPAVNHHSPLYSMEEDDEYNFDAQLTIDHTVNHYMKSGADRNKLVLGIPTYGRSYTLFNPLATELGSPADGPGEQGDSTREKGYLAYYEICENLQSDDWKVVQPNPSAMGPYAYKGNQWVSYDDMDIIKKKAQYVNDNGLGGIMFWAIDNDDFRGKCHGRPYPLIEAGKEAMLKGVKRSNNEIETTPVQNNRQSSRKRNRNRSKGNARGRTRT

TASTSTVVTTTTTTTTTTAAPLITPSYTTPEPPTTPDPGSDFKCKDEGFFPHPRDCKKYFWCLDSGPSNLGIVAHQFTCPSGLFF
NKAADSCDYARNVVCNKKSKSQGGSSSTLPPIKAATSSTTRFSTSPSTKLTTKLTTTTTTEPPPVLDDDDDDD

Sg-cht6-2
MNIRVKQPVIIGNCYRGQPNRLWEVFILKWFLVAVACLIAAGAVTVYLAHYFMKTRYTSTNVTGVTGQHSDLNTYKGQLQDM
GDGYSLFKQEDMTQICKTDELTGSQQMRKQSTKLVCYYTFPGPGGLVPDKIDPFLCTHINIAAVGINNSKLEPLCEERKEVIKSL
VGLKTRNKNLKVILSVIGMPGGFGDMVSKSSSRRMFIKDL

Sg-cht8-1
MSHFWLRLAVILGVSLSICGAEDKKVVCYHGSWSAYRNGNGRFEIEYIQPELCTHLIYTFVGITSAGEVRILDEWLDLPSGKNAY
NRFNALKSSNTKTLVAIGGWNEGSATYSAVMNDASLRAKFVQNVVNFVKTYGFDGFDLDWEYPANRGGSPGDLTAFVELIKE
LRTEFDKYGYLLTAAVGVGRYLIGTAYDVPQISKYLDFINLMTYDLHGSWDGKTGQNAPLYASSADKTEAERQLNVDSSVRYW
IQNGADPSKLVLGMGTYGRTFTLSSAANTGVGAPATAPGTNGPYTMESGMMGYNEICEKINAGGWTVVWDEEQKVPYAVNG
NQWIGYDNEESIRLKSQYVLDMGLAGGMIWSLETDDFKGLCGSKTYPLLSTINEVLRGITSTNSGSSSSSSSSSSSSSSSSSSSSSSSS
SSSSNTAASASSSSGVCSSAGYVRDPSDCGVFYLCTASGSGYTASKFTCPGDLVFDESSSACNYKSLVAC*

Sg-cht8-2
MSPFLSGLLLLLGVLNICGADEKKVVCYHGSWSAYRNGNGRFEIEYIRPELCTHMIYSFVGITSAGEVRILDEWLDLASGKNAYN
RFNKLKSSNTKTLVAIGGWNEGSATYSAVMNNAALRQKFVQNVVNFVKTYGFDGFDLDWEYPANRGGSPGDLRAYVELLKEL
RAEFDKHGFILSAAVGVGRYLIGSAYDVPQLSKYLDFINL

Sg-cht8-3
ESGMMGYNEICEKIKAGGWKVTWDDEQKVPYAVSGNQWVGYDNEESIKLKSQYVLDMGLGGGMIWSLETDDFKGVCGAGTF
PLLSAINQVLRGAAATSSAGSSTSGSSSGSSGSSSSSSASSGGSSSGTSSGSSTTSSGASADSSGSSASSGSSSSGSSATSVSSGSNSSG
VCNSAGYARDPSDCGV

Sg-idgf-1
MAELPLLLLLLAAAATCWTSAAALGATRVVCYLDGGALRRPEPHRMLVSEIEPSLTYCTHLIYGYATIDTDSYKAVPRHEGEGT
NYTSVVALKRRFPALNVLLSIGGGSADSGQREKYLHLLESDEHRRTFVKSAKDLLKQYHFDGIDIAWEFPMNKEKKERSTLGSF
WHGFKKVIGLAHSHKDEKADEHRREFSSLIQELKTSLKTENALLTLSVIPYINHTLYYDCSALSPHVDHLHLLAYDYHTPQRTP
NTADYPAPLYVAGKRDPDLTADGNVRWFLERGFPSRKIILGIPTFARTWKLDDDSRVSGVPPIEADGAGDTDNIANTAGIMAF
QTVCMLLPNAGNAGYKTTLSRVTDPTDRLGSYGFRLPSGEVTGLWVGYEDPDVAQYKAAYAKIKSLGGIAFSDLSLDDYHGICT
GDKYPIVRAGTLKLRYK*

Sg-idgf-2
MQSFARLLLLSACCWSAALAATTKVVCYFNTSALKRPESSRMLLSQIEPSFSYCTHLVVGYATINTETYKAVPPSEDEHTTYTNI
VALKRRFPSLKILLSIGGGAADTDTREKYFELLESDEHRTTFVSSAKSLLKQHGFDGIDIAWEFPKNKAKKDRGTFGSIWHGIKK
AVGAAHSHTDEKADEHKSQFSALIRELRTSLRNENALLTLSVIPYINQSLYYDPTALNQQIDELHVLAFDYRNPERDSQGGRLPC
AALPSRAEGLRPLGRREHPLVPRELIPS*

Sg-idgf-3
TDSYKAVPRYQDDTTKYTSLVALKERFPSLKVLLSIGGGGADADQRKKYLELLESDEHRRTFVDSVKELLQQNRFDGIDIAWEFPFSK
EKKDRGNVWHGVKKVLGYAHSHRDENPDEHRRQFSALIRELKSSLKTQNALLTLSVIPYINHSLYYDCASLSPEIDQLHLLAYDYHSP
TRTPKKADYPAPLYRAGERSADLTVDGNVRWFLEKGFPSRKIILGIPTFARTWKLTKDSRITGVPPIDADGPGVAGSIANISGLLAYQT
VCTLLPNDANAAYRTTLRRVTDPTDRLGSYGFRLPTREVSGLWVGYENQHSAEYKAAYARKKSLGGIAFSDLSLDDYNGVCTGEKFP
IVRAGTLKLLSTSV*

Figure S5. Amino acid sequences and conserved domains of *S. gregaria* chitin degrading enzymes.

(A) NAGs, (B) CHTs. Signal peptide and transmembrane region identified by Phobius (http://phobius.binf.ku.dk/index.html) and conserved domains identified by SMART (http://smart.embl-heidelberg.de/) are underlined and coloured. In (A) and (B): signal peptide: magenta, transmembrane region: dark blue. In (A): Glycohydro 20b2 domain (N-terminal domain of the eukaryotic beta-hexosaminidases): light green, Glyco hydro 20 domain (glycoside hydrolase

family 20 catalytic domain): grey. In (B): Glyco 18 domain (catalytic domain): light blue, Chitin-binding domain type 2 (ChBD2): green; catalytically critical consensus sequence in the Glyco 18 domain, FDG(L/F)DLDWE(Y/F)P, is highlighted in yellow and amino acid changes from the consensus are coloured in orange.

FIGURE S6

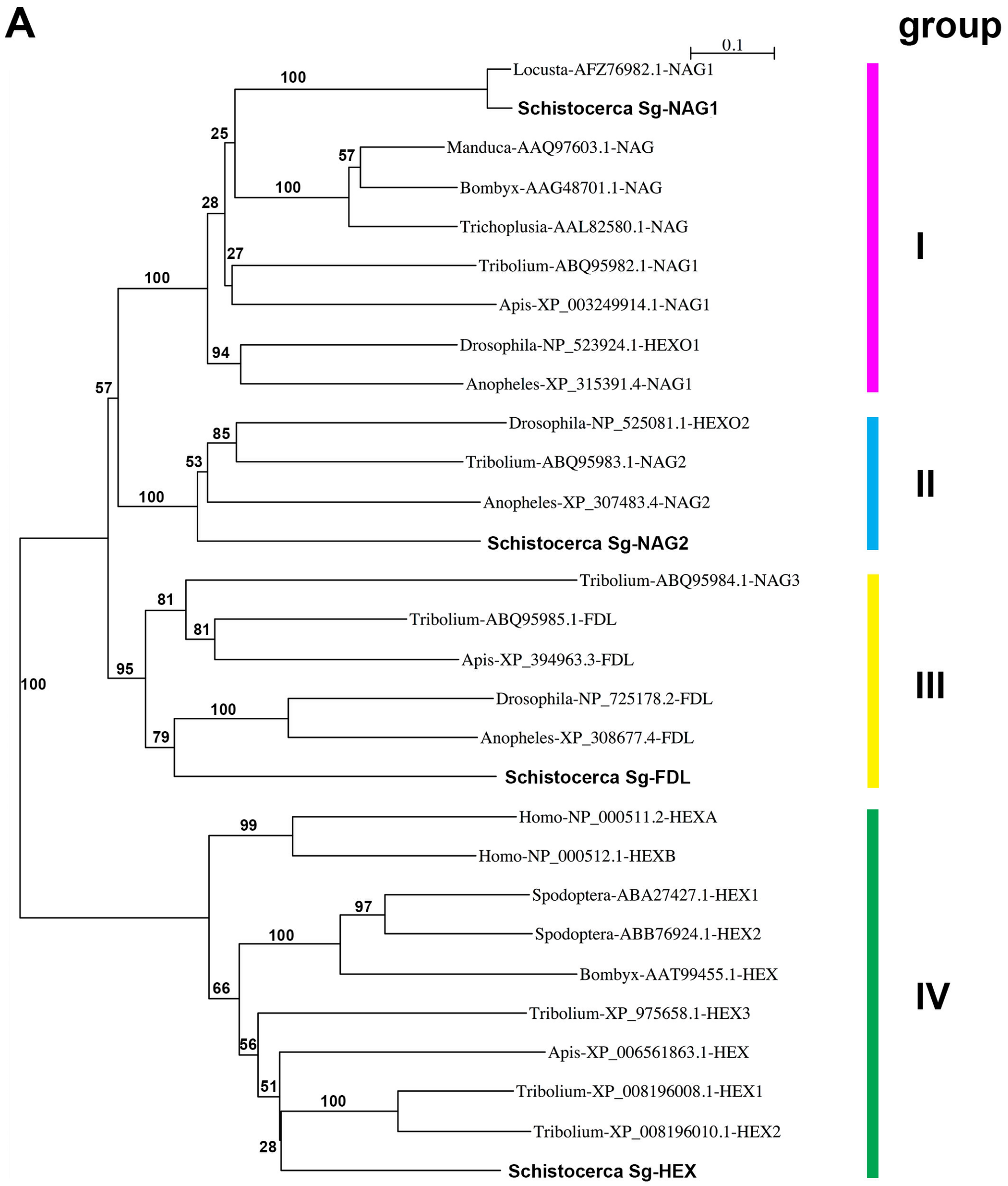
A
group
0.1
Locusta-AFZ76982.1-NAG1
Schistocerca Sg-NAG1
Manduca-AAQ97603.1-NAG
Bombyx-AAG48701.1-NAG
Trichoplusia-AAL82580.1-NAG
Tribolium-ABQ95982.1-NAG1
Apis-XP_003249914.1-NAG1
Drosophila-NP_523924.1-HEXO1
Anopheles-XP_315391.4-NAG1
Drosophila-NP_525081.1-HEXO2
Tribolium-ABQ95983.1-NAG2
Anopheles-XP_307483.4-NAG2
Schistocerca Sg-NAG2
Tribolium-ABQ95984.1-NAG3
Tribolium-ABQ95985.1-FDL
Apis-XP_394963.3-FDL
Drosophila-NP_725178.2-FDL
Anopheles-XP_308677.4-FDL
Schistocerca Sg-FDL
Homo-NP_000511.2-HEXA
Homo-NP_000512.1-HEXB
Spodoptera-ABA27427.1-HEX1
Spodoptera-ABB76924.1-HEX2
Bombyx-AAT99455.1-HEX
Tribolium-XP_975658.1-HEX3
Apis-XP_006561863.1-HEX
Tribolium-XP_008196008.1-HEX1
Tribolium-XP_008196010.1-HEX2
Schistocerca Sg-HEX
I
II
III
IV

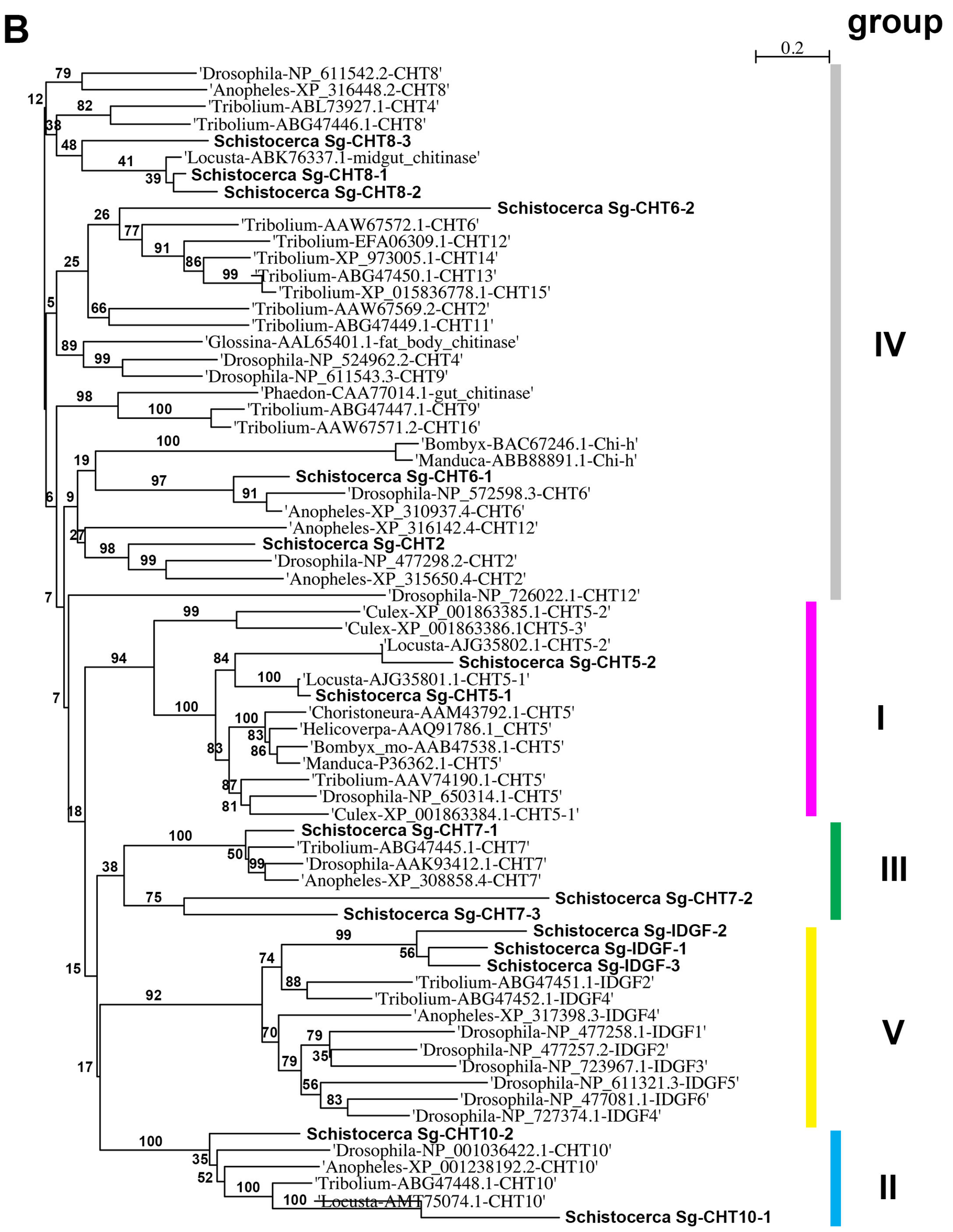

Figure S6. Phylogenetic trees of chitin degrading enzymes in *S. gregaria* and other insects.

(A) NAGs, (B) CHTs. *S. gregaria* sequences are in bold. Amino acid sequences were extracted from NCBI GenBank. The numbers above the branches are bootstrap support. The markers show a branch length. Both trees are unrooted. The tree in (A) was prepared using the SeaView software (version 4.6.1; Gouy et al, 2010; http://doua.prabi.fr/software/seaview): alignment with default parameters, tree using the Neighbor Joining method, Poisson distribution, 5000 bootstrap replicates. The tree in (B) was prepared using the CLC Sequence Viewer (version 7.8.1; https://www.qiagenbioinformatics.com/products/clc-sequence-viewer/): alignment with default parameters except gap open cost 3.0 and gap extension cost 3.0, tree using Neighbor Joining method, Kimura model, 1000 bootstrap replicates.

FIGURE S7

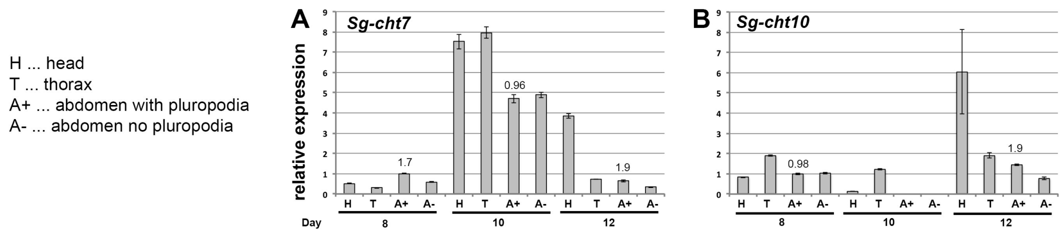

Figure S7. Real-time RT-PCR expression analysis of *Sg-cht7-1* and *Sg-cht10-1* on cDNA from parts of *S. gregaria* embryos.

cDNA was prepared from mRNAs isolated from parts of embryos at the age of 8, 10 and 12 days: H, head; T, thorax; A+, abdomen with pleuropodia; A-, abdomen without pleuropodia. Analysis of 3-4 technical replicates is shown. Expression in A+8 (abdomen with pleuropodia when they first become differentiated) was set as 1. Numbers above A+ expression is fold change from A- of the same age.

SUPPLEMENTARY REFERENCES

(for Additional file 1 and Additional file 2)

Angelini DR, Liu PZ, Hughes CL, Kaufman TC. Hox gene function and interaction in the milkweed bug Oncopeltus fasciatus (Hemiptera). Dev Biol. 2005;287:440-455.

Beermann A, Jay DG, Beeman RW, Hülskamp M, Tautz D, Jürgens G. The Short antennae gene of Tribolium is required for limb development and encodes the orthologue of the Drosophila Distal-less protein. Development. 2001;128:287-297.

Bennett RL, Brown SJ, Denell RE. Molecular and genetic analysis of the Tribolium Ultrabithorax ortholog, Ultrathorax. Dev Genes Evol. 1999;209:608-619.

Chintapalli VR, Wang J, Herzyk P, Davies SA, Dow JA. Data-mining the FlyAtlas online resource to identify core functional motifs across transporting epithelia. BMC Genomics. 2013;14:518.

Gouy M, Guindon S, Gascuel O. SeaView version 4: a multiplatform graphical user interface for sequence alignment and phylogenetic tree building. Mol Biol Evol. 2010;27:221-224.

Hughes CL, Kaufman TC. Hox genes and the evolution of the arthropod body plan. Evol Dev. 2002;4:459-499.

Kelsh R, Weinzierl RO, White RA, Akam M. Homeotic gene expression in the locust Schistocerca: an antibody that detects conserved epitopes in Ultrabithorax and abdominal-A proteins. Dev Genet. 1994;15:19-31.

Liu HW, Wang LL, Tang X, Dong ZM, Guo PC, Zhao DC, Xia QY, Zhao P. Proteomic analysis of Bombyx mori molting fluid: Insights into the molting process. J Proteomics. 2018;173:115-125.

Prpic NM, Wigand B, Damen WG, Klingler M. 2001. Expression of dachshund in wild-type and Distal-less mutant Tribolium corroborates serial homologies in insect appendages. Dev Genes Evol. 2001;211:467-477.

Tear G, Akam M, Martinez-Arias A. Isolation of an abdominal-A gene from the locust Schistocerca gregaria and its expression during early embryogenesis. Development. 1990;110:915-925.

Wei Z, Yin Y, Zhang B, Wang Z, Peng G, Cao Y, Xia Y. Cloning of a novel protease required for the molting of Locusta migratoria manilensis. Dev Growth Differ. 2007;49:611-621.

Zhang J, Lu A, Kong L, Zhang Q, Ling E. Functional analysis of insect molting fluid proteins on the protection and regulation of ecdysis. J Biol Chem. 2014;289:35891-35906.

Zhang H, Shinmyo Y, Mito T, Miyawaki K, Sarashina I, Ohuchi H, Noji S. Expression patterns of the homeotic genes Scr, Antp, Ubx, and abd-A during embryogenesis of the cricket Gryllus bimaculatus. Gene Expr Patterns. 2005;5:491-502.
